# Spatiotemporal patterns of variability in the abundance and distribution of winter-spawned pelagic juvenile rockfish in the California Current

**DOI:** 10.1371/journal.pone.0251638

**Published:** 2021-05-27

**Authors:** John C. Field, Rebecca R. Miller, Jarrod A. Santora, Nick Tolimieri, Melissa A. Haltuch, Richard D. Brodeur, Toby D. Auth, E. J. Dick, Melissa H. Monk, Keith M. Sakuma, Brian K. Wells

**Affiliations:** 1 Fisheries Ecology Division, Southwest Fisheries Science Center, National Marine Fisheries Service, National Oceanic and Atmospheric Administration, Santa Cruz, CA, United States of America; 2 Institute of Marine Sciences, Fisheries Collaborative Program, University of California, Santa Cruz, CA, United States of America; 3 Conservation Biology Division, Northwest Fisheries Science Center, National Marine Fisheries Service, National Oceanic and Atmospheric Administration, Seattle, WA, United States of America; 4 Fisheries Research, Analysis and Monitoring Division, Northwest Fisheries Science Center, National Marine Fisheries Service, National Oceanic and Atmospheric Administration, Seattle, WA, United States of America; 5 Fish Ecology Division, Northwest Fisheries Science Center, National Marine Fisheries Service, National Oceanic and Atmospheric Administration, Newport, OR, United States of America; 6 Pacific States Marine Fisheries Commission, Newport, OR, United States of America; Havforskningsinstituttet, NORWAY

## Abstract

Rockfish are an important component of West Coast fisheries and California Current food webs, and recruitment (cohort strength) for rockfish populations has long been characterized as highly variable for most studied populations. Research efforts and fisheries surveys have long sought to provide greater insights on both the environmental drivers, and the fisheries and ecosystem consequences, of this variability. Here, variability in the temporal and spatial abundance and distribution patterns of young-of-the-year (YOY) rockfishes are described based on midwater trawl surveys conducted throughout the coastal waters of California Current between 2001 and 2019. Results confirm that the abundance of winter-spawning rockfish taxa in particular is highly variable over space and time. Although there is considerable spatial coherence in these relative abundance patterns, there are many years in which abundance patterns are very heterogeneous over the scale of the California Current. Results also confirm that the high abundance levels of YOY rockfish observed during the 2014–2016 large marine heatwave were largely coastwide events. Species association patterns of pelagic YOY for over 20 rockfish taxa in space and time are also described. The overall results will help inform future fisheries-independent surveys, and will improve future indices of recruitment strength used to inform stock assessment models and marine ecosystem status reports.

## Introduction

Along the US West Coast, the genus *Sebastes*, commonly known as rockfish, is a highly speciose group of over 60 species that are generally long-lived and slow growing, and occupy a range of habitats ranging from the nearshore to the continental slope [1, 2]. While most adults are associated with benthic habitat, many of the more abundant species have midwater habitat associations. All *Sebastes* produce live larvae, and the most abundant populations tend to concentrate their spawning in winter months, with widely dispersed pelagic juvenile stages which in turn are key components of pelagic marine food webs [1, 3]. Recruitment, or cohort (year-class) strength for most well studied populations is highly variable, often with orders of magnitude separating strong from weak year classes [4, 5]. As with many marine fishes, observed year-to-year recruitment estimates for *Sebastes* species do not tend to be strongly related to spawning biomass or spawning output [6–8]. Most species are targeted by a wide range of commercial and recreational fisheries, and seven U.S. rockfish stocks were formally declared overfished in the late 1990s and early 2000s, with many others were retroactively assessed to have been below target levels during this period. Since that time, a better understanding of *Sebastes* life history, improved assessment models, more effective management measures (including vessel buybacks and area closures), and ocean conditions favoring strong recruitment have led to all but one of those stocks recovering to levels of abundance above their management targets [9–11].

For most marine fishes, it is generally accepted that the relative magnitude of year class strength is set during the first 30 days of life, with environmentally driven density-independent processes proving more important than density-dependent factors such as spawning output [6, 12, 13]. This logic has generally held true for rockfishes in the California Current [4, 14, 15], although density-dependent processes may scale year-class strength in later life-history stages [16, 17]. The considerable longevity of most rockfishes (and many other groundfish) is thought to be an adaptation to an unpredictable environment and unfavorable recruitment conditions that may keep recruitment levels low for long time periods [18, 19]. When strong year classes do occur, they can later lead to short-term spikes in catch rates of smaller, younger individuals, potentially complicating quota-based management efforts if the increased availability to fisheries is not forecast by the stock assessment models that inform management. Monitoring the abundance and distribution of juveniles can provide recruitment indices to inform stock assessment models, by providing data on the relative magnitude of incoming year classes subsequent to the larval and late-larval stages during which most density-independent mortality takes place [6, 20, 21].

**Fig 1:**
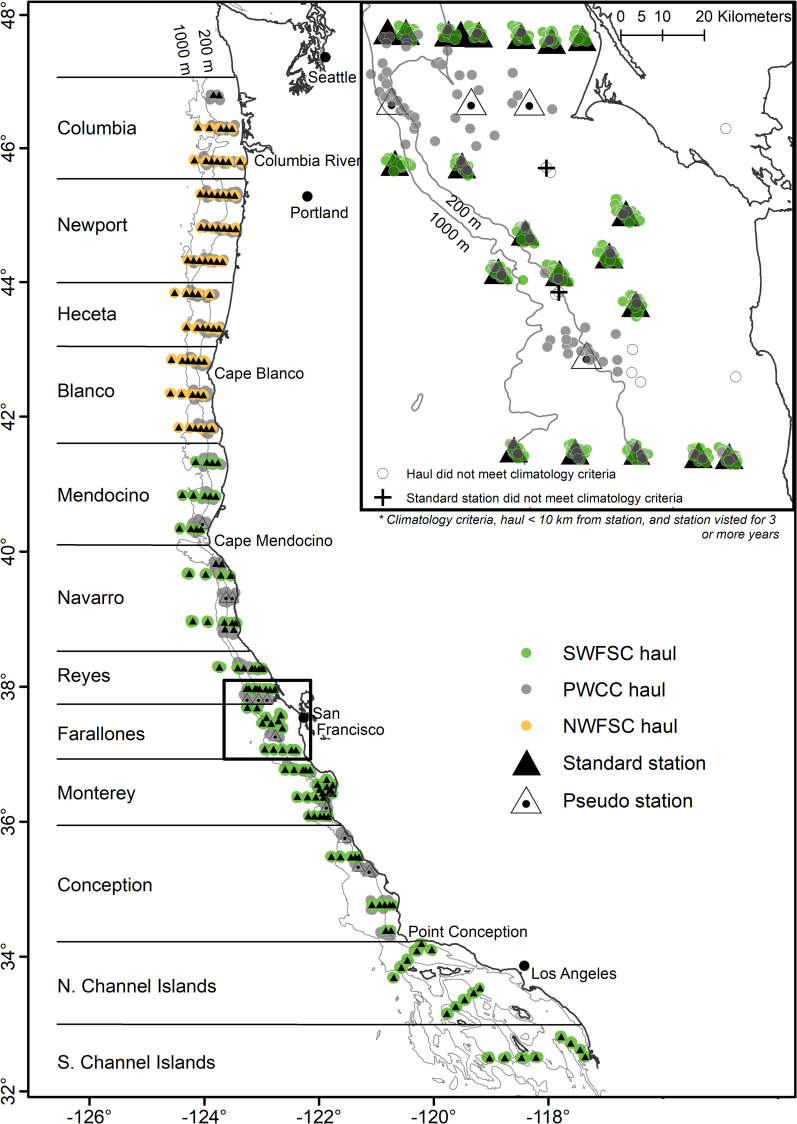
Station map, including fixed stations, pseudo-stations, regions, and major biogeographic boundaries.

From 1983 through 2019, data on the abundance of age-0 pelagic juvenile rockfishes (*Sebastes* spp.) were collected in annual midwater trawl surveys conducted in late spring (May and June). These surveys provide indices of abundance for year-class strength in stock assessments for winter-spawning rockfish, with the goal of helping to inform forecasts of population trajectories and yield. The survey data also informs studies related to the oceanographic processes thought to be the drivers of variable year-class strength, as well as the ecosystem consequences that result from variable abundance of young-of-the-year (YOY) rockfish and other forage species [4, 11, 22, 23]. Data are publicly available on the NOAA ERDDAP server [24]. For the first 20 years of the survey, the spatial footprint was central California; from the southern end of Monterey Bay to just north of Point Reyes (Fig 1). Data were collected over a larger area beginning in 2001, in order to account for the broader spatial scale at which stocks are distributed and assessed, and resolve the effect of oceanographic drivers on interannual regional variability in distribution patterns. For example, the strong 1999 year class became apparent in virtually all monitored West Coast adult groundfish populations during the early 2000s, yet catch rates in the 1999 pre-recruitment survey were closer to long-term average levels, such that the spatially-limited survey failed to detect and predict high recruitment success for that important year class [4, 15].

An initial evaluation of the first few years of species catch abundances from the expanded survey range confirmed relative abundance patterns were spatially variable among years along the U.S. West Coast, likely in response to spatially variable ocean productivity and transport patterns. Specifically, catches from (core) central California region were very low during unusual climate and upwelling conditions in 2005 and 2006, but higher in the northern and southern California Current regions. The 2005–2006 time period was also associated with unusually low primary and secondary productivity in central California, which in turn led to salmon run failures, seabird die-offs and other higher trophic level impacts [25–27]. After finding strong heterogeneity of pelagic YOY rockfish abundance in those years, a recommendation was made to only use indices informed by data of comparable spatial scale to the stock assessment in which the index was used, which is variable, but generally covers much or all of the U.S. West Coast [28]. Analysis of catch from the first five years of coastwide data confirmed the patterns observed in 2005 and 2006, but indicated that those years were unusual relative to catch rates and distributions from the rest of the 2004–2009 time period, likely in response to unusual wind forcing and subsequent ocean advection patterns. Specifically, the authors found that dominant wind patterns were either reversed or more variable for much of the central California to Central Oregon region during February of 2005 and 2006 [28].

Environmental drivers of year to year abundance patterns of pelagic YOY rockfish catches have been rigorously explored elsewhere for this [4, 11] and other YOY rockfish datasets [29–31]. Oceanographic features are clearly drivers of observed spatial distribution patterns at finer spatial scales, as has been shown for both larval [32] and juvenile rockfishes [33, 34]. Previous analyses have also shown a high degree of synchrony among species within the central California survey region [4], as well as mesoscale synchrony in overall abundance among shelf, slope and canyon pelagic habitats [22]. While mid-trophic level forage communities are generally highly variable from year to year [22, 23, 29], there is some general coastwide spatial coherence in major changes in the forage assemblage in response to large scale oceanographic drivers such as the El Niño/Southern Oscillation (ENSO) and marine heatwaves [35, 36]. The expanded coastwide data collections continue to be used to provide recruitment indices for stock assessments of commercially and ecologically important rockfish stocks, including the those for bocaccio (*Sebastes paucispinis*), chilipepper (*S*. *goodei*), blue/deacon (*S*. *mystinus/ S*. *deacanus*), canary (*S*. *pinninger*), and widow (*S*. *entomelas*) rockfish [37–41]. However, constraints on survey vessel resources and other factors have resulted in spatiotemporally variable survey effort since 2001, making the development of truly coastwide indices for some years impossible. Consequently, data from many partially sampled years are excluded from some stock assessments, contributing to greater uncertainty in assessment forecasts.

As of 2019, survey data are coastwide, from the southern Channel Islands near the U.S./Mexico border to the waters north of the Columbia River, off of Grays Harbor, Washington, for 13 (2004–09, 2013–19) of the past 19 years of expanded area survey extent. This analysis examines the year to year distribution patterns observed in the coastwide data in greater detail, in order to quantify and characterize the spatial climatology (the long-term average spatial abundance pattern) of pelagic YOY rockfish distribution, and to evaluate the hypothesis that the abundance of pelagic YOY covaries over the scale of the California Current from year to year. We also characterize the species association patterns across space, to better understand whether multispecies pre-recruit indices may be appropriate for sparsely sampled species that covary strongly over space and time, but lack sufficient data to develop species specific indices [as in 5]. The results will inform general patterns in pelagic juvenile abundance, as well as an improved understanding of coherence in catch rates over space and time, which in turn will help to inform the development of pelagic YOY abundance indices (inclusive of sampling and survey effort considerations) for stock assessments. These findings will also inform ecosystem studies of the role of pelagic YOY in regional food webs, and improve our understanding of geospatial and temporal variability of oceanographic processes and drivers of productivity throughout the California Current, particularly with respect to recruitment of commercially important species. Ongoing and future efforts will continue to explore the mechanistic drivers (e.g., environmental and oceanographic forcing) of variable distribution and abundance of rockfish (and other forage taxa) throughout the California Current.

## Methods

### Surveys design and coverage

Three complementary survey efforts have been integrated into this analysis. These include: (1) the NOAA Fisheries Rockfish Recruitment and Ecosystem Assessment Survey (RREAS), conducted off central California annually between 1983 and 2003 and off most California waters from 2004–2019 [42]; (2) the Pacific Whiting Conservation Cooperative/Northwest Fisheries Science Center (PWCC/NWFSC) cooperative survey, conducted from the Pacific Northwest down to central California in 2001–09 [28, 43]; and (3) the NOAA Fisheries pre-recruit survey, conducted off of Oregon and Washington in most years since 2011 [23]. All surveys used the same fishing gear, sampling methods, and processing protocols, and thus are treated as comparable in published analysis [28, 29, 44, 45] and stock assessments [37, 38]. All three surveys use a modified-Cobb midwater trawl with a 26-m headrope and a 9.5-mm cod-end liner that retains epipelagic micronekton. Net mensuration data from time-depth recorders and SIMRAD ITI acoustic sensors confirm that the height and width of the net average 12 m each when the net is fishing, resulting in the net sampling area of approximately 144 m^2^. The target depth of the headrope is 30 m for standard hauls, except for a small number of nearshore stations (< 60-m bottom depth), where the net is fished at 10 m to avoid bottom contact. Previous studies have demonstrated that these depths are appropriate for the most commonly encountered rockfish taxa in these surveys [46]. Tows are standardized by deploying ~85 m of trawl warp and adjusting the ship’s speed in real time to maintain the headrope depth at 30 m, which results in a ship speed of approximately 2.0 knots (3.7 km/hr). The tow duration is 15 minutes from the time the headrope reaches the target depth. Upon completion of a trawl, the contents of the cod-end are immediately sorted and enumerated to the lowest possible taxon [23, 42]. Rockfish < 20 mm (~50 days old) in standard length are removed from the analysis due to low selectivity of net mesh [14].

In the case of the RREAS and the Pre-recruit survey, the survey designs are based on a fixed-station sample grid that has been modified over time to improve and optimize sampling efficiency [23, 42]. Stations are grouped into regional clusters or lines of four to seven stations, such that it is typically possible sample all stations in a line or cluster during a night of sampling (recognizing that “nights” in late Spring are quite short). The target organisms, pelagic juvenile rockfish, avoid the sampling gear during the day, thus all sampling is done during hours of complete darkness, which can be as little as 7–8 hours per period of darkness in late Spring, depending upon the latitude and calendar day. Consequently, the distance between stations in a given line or cluster is constrained spatially by the distance that the survey vessel is able to transit between the stations in a given night of sampling. Ideally, station lines or clusters are sampled 2 to 3 times in a given year to account for short-term temporal variation in catch rates, although the PWCC/NWFSC survey from 2001–2009 conducted a single north to south sweep of the coast each year. Hauls from both NOAA Fisheries sampling programs that did not comport to a standard station or standard haul parameters (e.g., those done in daytime, or at deeper depths, or aborted due to high jellyfish abundance or other factors), were excluded from the analysis. Stations that are no longer actively sampled but were sampled for at least three years during the climatology period were included in the spatial climatology analysis, whereas any station sampled less frequently than three of the climatology years was excluded from the mapping effort. This pre-processing resulted in the exclusion of only a small number of hauls (52) from the spatial climatology. A small number of hauls (66) from north of the Columbia region, which were only sampled during five years of effort, were excluded from both the mapping and the temporal variability analysis.

The PWCC/NWFSC sampling conducted in 2001–09 did not occupy distinct stations, but conducted a series of onshore/offshore transects, starting in northern latitudes and transiting south. Trawls were generally clustered close to each other from year to year but not repeat pre-determined stations from year to year. However, most (73%) PWCC hauls could be associated with a nearby NOAA Fisheries station. When clusters of PWCC hauls were more than 10 km from a defined station from one of the other two surveys, a pseudo-station was created in the centroid of the distribution of that cluster. We defined 11 such pseudo-stations that consisted of 192 total hauls (each pseudo-station had between 8 and 28 hauls). The remaining PWCC hauls, those with too few observations or years sampled to associate with a cluster, and those more than 10 km from an existing NOAA Fisheries station (as well as those that did not meet the performance parameters applied to the SWFSC and NWFSC surveys), were excluded from the spatial climatology and mapping analysis. Survey stations and pseudo-stations are shown in Fig 1, along with lines demarcating the twelve different geographic regions that we grouped adjacent lines within for later spatial analyses. These regions are based on both biogeographic boundaries as well as the approximate maximum distance that an oceanographic research vessel could transit between consecutive nights of sampling. The raw data, including haul date, starting latitude and longitude of each tow, bottom depth at tow start locations, region, area and station assignments, and the catch by species, is included as a supplementary data file (YOYrockfish20012019.csv).

### Spatial distribution patterns

We developed a 13-year spatial catch rate climatology for pelagic YOY rockfish in the coastal waters of the California Current, from the southern Channel Islands near the U.S./Mexico border to the waters north of the Columbia River (approximately 32 to 47° N). A total of 183 stations met the climatology criteria (of being sampled for at least three of the sampling years), resulting in 1561 station-by-year rockfish catch observations. Given that catch rates vary by orders of magnitude from year to year and station to station, catches were log-transformed (ln [catch+1]) prior to analysis and transformed catches were averaged for station-year combinations that had > 1 haul per year for mapping purposes. We mapped distribution patterns for the 10 most frequently occurring rockfish species in the historical central California region [4], all rockfish species combined, and rockfishes broadly aggregated as either targets of commercial and recreational fisheries or forage species important to food webs but too small to be of commercial or recreational interest. The fishery target taxa included bocaccio, chilipepper, widow, canary, and yellowtail (*S*. *flavidus*) rockfishes, while forage taxa included shortbelly (*S*. *jordani*), squarespot (*S*. *hopkinsi*), stripetail (*S*. *saxicola*), and halfbanded (*S*. *semicinctus*) rockfishes. We also mapped the ratio of target to forage species abundance. Data were interpolated using Inverse Distance Weighting kriging. The spatial neighborhood was set to five: the approximate number of stations in a typical survey transect. We also evaluated inter-annual spatial anomalies in catch rates of all pelagic juvenile rockfish by calculating the z-scores of the log-transformed data. The inter-annual spatial anomalies were plotted using the same Inverse Distance Weighting approach as the spatial climatologies, to provide a visual interpretation of spatial variability in relative abundance patterns.

### Regional temporal variability and synchrony

To evaluate the degree of spatial coherence over time, standardized summaries of regional catch rate estimates (among all rockfish taxa combined) were developed using delta-GLM (otherwise known as hurdle) models [47, 48]. Individual hauls were the input data, with the year effects being the parameter of interest, and other covariates including stations or lines, inshore/offshore depth bins, and calendar date effects [4, 11]. Thus, for each region, abundance of positive observations was estimated as

$$log(abundance)=\mu +Y_{i}+D_{j}+S_{k}+P_{l}+\varepsilon$$ (1)

where μ is the average log(abundance), Y_*i*_ is a year effect, D_*j*_ is a depth bin effect, S_*k*_ is a station or line (region) effect, P_*l*_ is a time period (Julian day, ten day bins) effect and *ε* is a normal error term with mean zero and variance *σ*^2^. Factors were included or excluded for each model based on whether they met Akaike Information Criteria (AIC). The same covariates were used to estimate the probability of a positive tow using a logit link function.

We then used dynamic factor analysis (DFA) [49, 50] to evaluate the presence of common trends among regions through 2001 to 2019. DFA is analogous to principal components analysis (PCA), in that it is a dimension-reduction technique that identifies common patterns among a group of variables. However, in DFA, time series are modeled as a linear combination of latent trends, such that *x_t_* is function of *x*_*t*−1_ [49, 50]. These trends reflect the shared temporal variation among the time series (here relative catch rates by regions, *y*_i_) and error terms that are specific to populations:

$$y_{i,t}=Zx_{i,t}+v_{i,t}.$$ (2)

The *Z* matrix contains the factor loadings, and the residual error is *v_i,t_* ~ MVN (0,*R*), where *R* is the variance-covariance matrix. The latent trends (*x_t_*) are a function of *x*_*t*−1_ with noise component (w):

$$x_{i,t}=\phi x_{i,t-1}+w_{i,t}\;and\;w_{i,t}∼MVN(0,1).$$ (3)

When *ϕ* approaches 1.0 the the trend behaves as a random walk. When *ϕ* approaches zero the trend behaves as white noise. DFA can address missing data through the use of a Kalman Filter [49, 50]. This is relevant to the current analysis due to gaps in the regional data in some years.

Prior to analysis the time series were standardized (z-scored). Both zeros and year-area combinations with no data were converted to null values, and were subsequently predicted by the model using Kalman Filters [49, 50]. We ran a total of 18 DFA models allowing the number of trends to vary between one and three and allowing either a diagonal and equal R (~homogeneous observation variance) or a diagonal and unequal R. We also estimated separate *ϕ*’s for each region (diagonal and unequal), one overall ø (diagonal and equal), or set *ϕ* = 1.0. We compared models by examining the delta Akaike Information Criteria corrected (AICc) values and model weights [51].

### Species assemblage patterns

We used the species-specific catches from the climatology years to evaluate spatial organization of the juvenile rockfish assemblage by quantifying the dissimilarities between the species composition in the sample units (i.e., individual hauls) using non-metric multi-dimensional scaling (NMDS) [52]. This approach evaluates the relationships among taxa by arranging objects in a low-dimensional (i.e., 2D or 3D) ordination space so that the inter-object distances (i.e., species values) in the input similarity matrix have the same rank order, with the measure of this distance termed stress. We excluded all rockfish not identified to the species level, as well as those taxa occurring in < 1% of hauls, leaving 20 species in the analysis. We used only the climatology years with full spatial coverage (2004–09 and 2013–19), and we excluded all hauls with fewer than three species, as well as 29 outlier hauls > 2 standard deviations from the overall mean distance, leaving 662 hauls for the NMDS [52]. A fourth-root transformation was applied, as rockfish catch rates spanned several orders of magnitude. The NMDS was conducted using the *vegan* package in R [53]. To explore key habitat features that relate to the assemblage patterns observed, Generalized Additive Models (GAMs) were fit to latitude contours with *ordisurf* in the *vegan* package, using the optimal number of knots (edf = 15.36, *p* value < 0.001).

## Results

When all standard hauls meeting good performance criteria were combined, the result was 3,839 hauls conducted between 2001 and 2019, with 2,344 from the RREAS (all years), 1183 from the PWCC/NWFSC survey (2001–09), and 312 from the NWFSC pre-recruit survey (2011, 2013–19). Of these, 66 hauls were taken north of the Columbia region, and those were excluded from further analysis. The number of hauls per climatology year ranged from 106 in 2017 to 323 in 2006, with an overall average of 222 per year (Table 1). In all of those hauls, a total of 272,360 pelagic juvenile rockfish were caught (excluding those < 20 mm), representing 33 rockfish species or taxonomic groupings. Shortbelly rockfish, a species of no current commercial importance but critically important in food webs, comprised 63% of the total catch during this time period, and other forage species, particularly stripetail, squarespot, and halfbanded rockfish, comprised an additional 15% of the catch (when unidentified catches were excluded). Commercial and recreational target species represented the remaining 22% of the catch that was identifiable to the species level. Common and scientific names of all species encountered, along with the number caught, their designation as either a target or forage species, and their mean asymptotic length (L_infinity_) based on their growth curve, which is used in later analyses, are also reported (S1 Table). Widow and chilipepper rockfish were the most frequently occurring of the commercially important species, representing slightly over 6% and slightly over 5% of the total catch, respectively, followed by yellowtail, bocaccio, blue/deacon, and canary rockfish, which ranged from 0.5 to 2% of the total catch. Data are included as S1 Appendix, which includes key haul information (year, date, latitude, longitude, station, region) as well as catch of YOY rockfish at the lowest taxonomic resolution available.

**Table 1:** Total number of successful hauls by region and year (2001–2019).

	01	02	03	04	05	06	07	08	09	10	11	12	13	14	15	16	17	18	19
SCI				9	15	16	12	15	8	7		4	8	6	15	13	8	12	8
NCI				15	18	15	16	15	8	12		11	15	8	23	18	9	17	13
Conception	15	20	16	27	27	41	18	22	20	5	2	7	18	22	21	21	9	17	20
Monterey	59	49	64	60	73	53	61	24	49	41	27	45	39	36	35	42	15	26	34
Farallones	35	31	43	49	37	35	52	23	50	33	15	22	22	29	34	23	20	21	11
Reyes	18	18	26	22	21	45	31	18	27	19	15	5	16	28	10	12	8	15	6
Navarro	10	15	15	24	27	34	32	31	25	16	10		11	11	14	4	13	12	7
Mendocino	15	15	16	15	15	16	15	13	17		18		14	11	14	3	11	11	4
Blanco	13	15	15	15	15	15	18	18	17		17		11	12	12	12	3	8	7
Heceta	11	10	10	10	10	10	11	10	11		12	3	10	8	8	8	2	6	7
Newport	4	5	5	22	15	17	16	15	17		15	4	12	12	11	13	6	10	12
Columbia				10	15	16	18	15	18		15		8	4	8	9	2	8	8
Total	180	178	210	278	288	313	300	219	267	133	146	101	184	187	205	178	106	163	137

### Spatial distribution patterns

**Fig 2:**
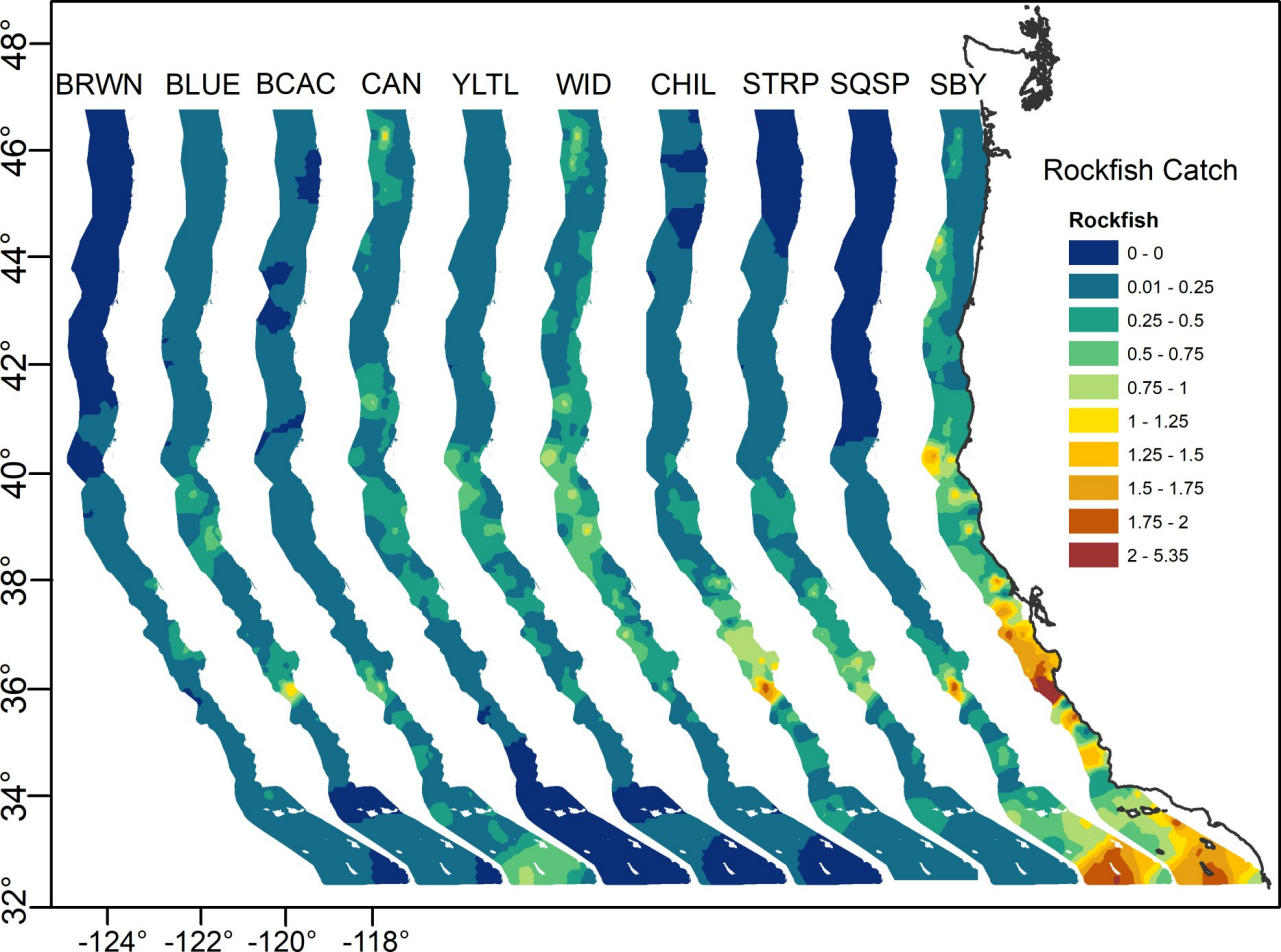
Mean log transformed CPUE of YOY rockfish by species (and for all species, “All Rf”) for all climatology years (2004–2009, 2013–2019). Common names as follows, BRWN = Brown, BLUE = Blue/Deacon, BCAC = Bocaccio, CAN = Canary, YLTL = Yellowtail, WID = Widow, CHIL = Chilipepper, STRP = Stripetail, SQSP = Squarespot, SBY = Shortbelly.

In general, the presence of pelagic juvenile rockfish during the timing of this survey was greatest in the southern and northern Channel Islands and the central California regions, where 60–85% of tows caught at least one individual (Table 2). Catches tended to be lower in the Pacific Northwest, particularly in the regions north of Cape Blanco, where only 32–37% of tows encountered pelagic juvenile rockfishes. These patterns are also visible in the catch rate maps (Fig 2), which indicate that when averaged across all climatology years, catches were greater in Southern California Bight (SCB) and central California waters. Distinct spatial gradients are present at the species level, with northern species such as widow, canary, and yellowtail rockfish extremely rare south of Monterey Bay, and southern species such as bocaccio, shortbelly, and squarespot rockfish dropping off sharply in waters north of San Francisco. Interestingly, several species for which abundance is generally considered to be far greater in northern waters, such as widow, yellowtail, and canary rockfish, had their highest catch rates off of central California.

**Table 2:** The frequency of occurrence (climatology years only) of positive tows by rockfish species and region, for the historical “top ten” rockfish taxa, as well as several others of commercial significance (black rockfish, cowcod, and darkblotched rockfish) and pooled groups (for scientific names and group designations see S1 Table).

	Southern Channel Islands	Northern Channel Islands	Conception/Big Sur	Monterey Bay	Gulf of the Farallones	Point Reyes	Navarro	Cape Mendocino	Cape Blanco	Heceata	Newport	Columbia
All rockfish	0.85	0.63	0.58	0.63	0.73	0.6	0.53	0.41	0.41	0.32	0.37	0.32
Forage taxa	0.76	0.53	0.53	0.56	0.6	0.44	0.35	0.26	0.21	0.12	0.09	0.07
Target taxa	0.78	0.52	0.49	0.55	0.62	0.5	0.44	0.36	0.35	0.25	0.35	0.31
*Squarespot*	0.54	0.29	0.19	0.15	0.08	0.04	0.03	0.01	0	0	0	0
Bocaccio	0.37	0.13	0.18	0.09	0.09	0.06	0.04	0.01	0.03	0.01	0.04	0.03
Cowcod	0.06	0.09	0.08	0.06	0.04	0.02	0	0	0	0	0	0
*Shortbelly*	0.54	0.36	0.43	0.48	0.53	0.35	0.23	0.21	0.17	0.1	0.09	0.07
Brown	0.01	0.07	0.04	0.12	0.11	0.05	0.01	0.01	0	0	0	0
*Stripetail*	0.08	0.11	0.2	0.27	0.3	0.22	0.18	0.08	0.06	0.03	0.01	0
Chilipepper	0.05	0.08	0.23	0.33	0.37	0.28	0.14	0.09	0.08	0.03	0.03	0.01
Blue/Deacon	0.01	0.01	0.05	0.15	0.17	0.16	0.13	0.08	0.06	0.04	0.07	0.06
Widow	0	0.02	0.08	0.22	0.26	0.21	0.2	0.25	0.13	0.1	0.12	0.18
Black	0	0	0	0.03	0.09	0.08	0.09	0.03	0.04	0.03	0.03	0.05
Yellowtail	0	0.01	0.05	0.13	0.2	0.19	0.18	0.12	0.1	0.08	0.08	0.09
Canary	0	0	0.01	0.07	0.22	0.22	0.2	0.14	0.19	0.09	0.13	0.16
Darkblotched	0.03	0	0.01	0.02	0.03	0.03	0.05	0.03	0.04	0.09	0.17	0.12

Colors indicate more (green) or less (red) frequently occurring observations, the species whose names are in normal font are commercial (or target) taxa, the species whose names are in italics are included in the “forage taxa” designation.

**Fig 3:**
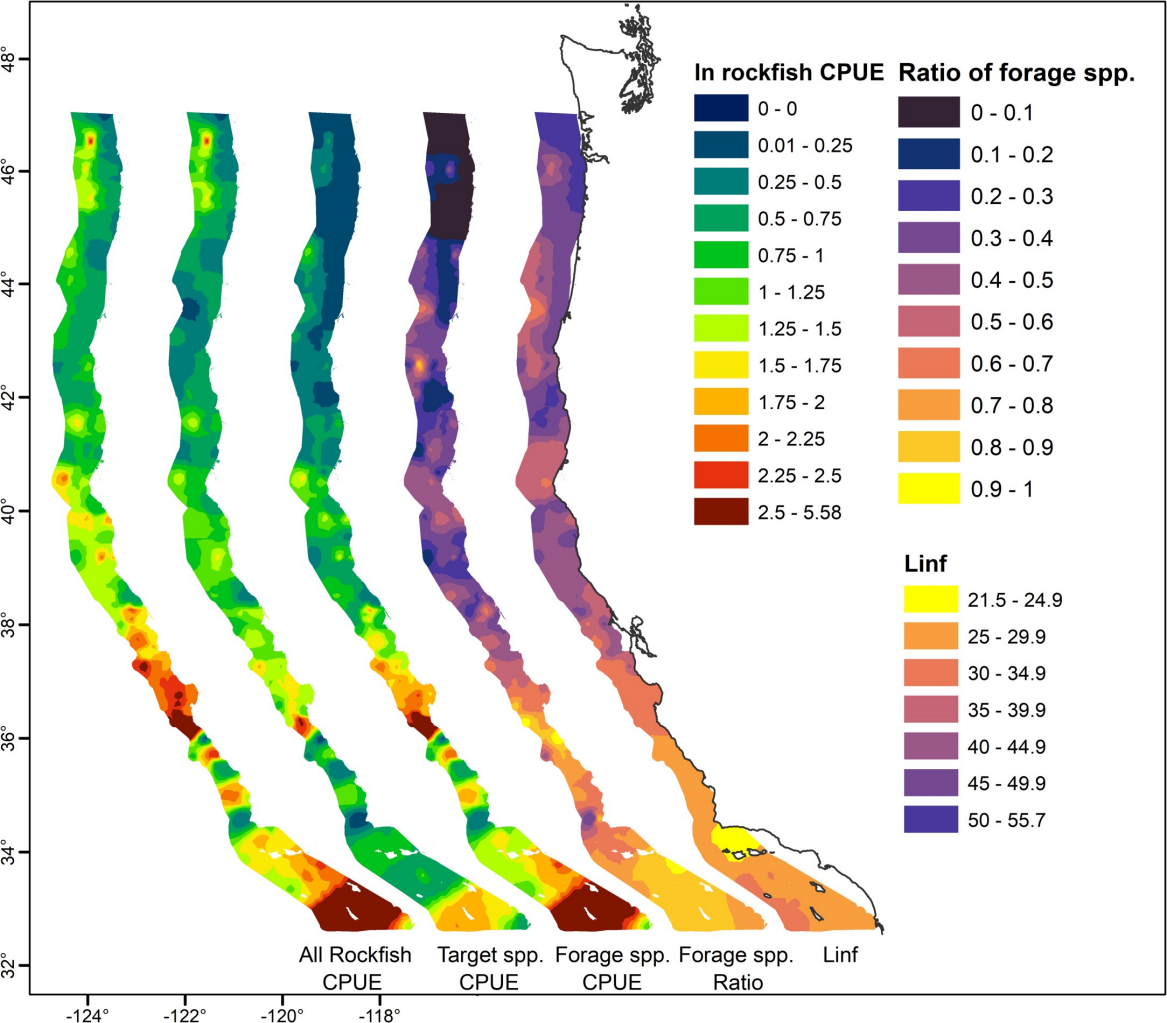
Climatological distribution of pelagic YOY rockfish when target or non-target (forage) species are pooled, showing that the abundance is dominated by non-commercially important taxa in southern waters. Map on far right provides catches weighted by the estimated L_infinity_ (theoretical maximum size) of adults (excluding mixed or unidentified taxa).

When species are pooled into broad categories (target vs. forage species), it becomes clear that the forage taxa are key drivers of the regional hotspots of abundance in central and southern California (Fig 3). While the commercially important taxa tend to be somewhat more abundant in southern and central California waters, their abundance is generally comparable across regions throughout California Current waters, whereas the forage taxa become significantly less abundant north of Cape Mendocino. This pattern is more evident when the fraction of the total rockfish catch assigned to each category is considered, as well as when total catches are scaled by the L_infinity_ estimate, where L_infinity_ is the asymptotic maximum size of adults based on the von-Bertalanffy growth equation (sources for each L_infinity_ estimate are reported in S1 Table) for each species, indicating a clear trend of greater abundance of taxa with larger maximum body size of *Sebastes* in more northern waters.

### Regional temporal variability and synchrony

**Fig 4:**
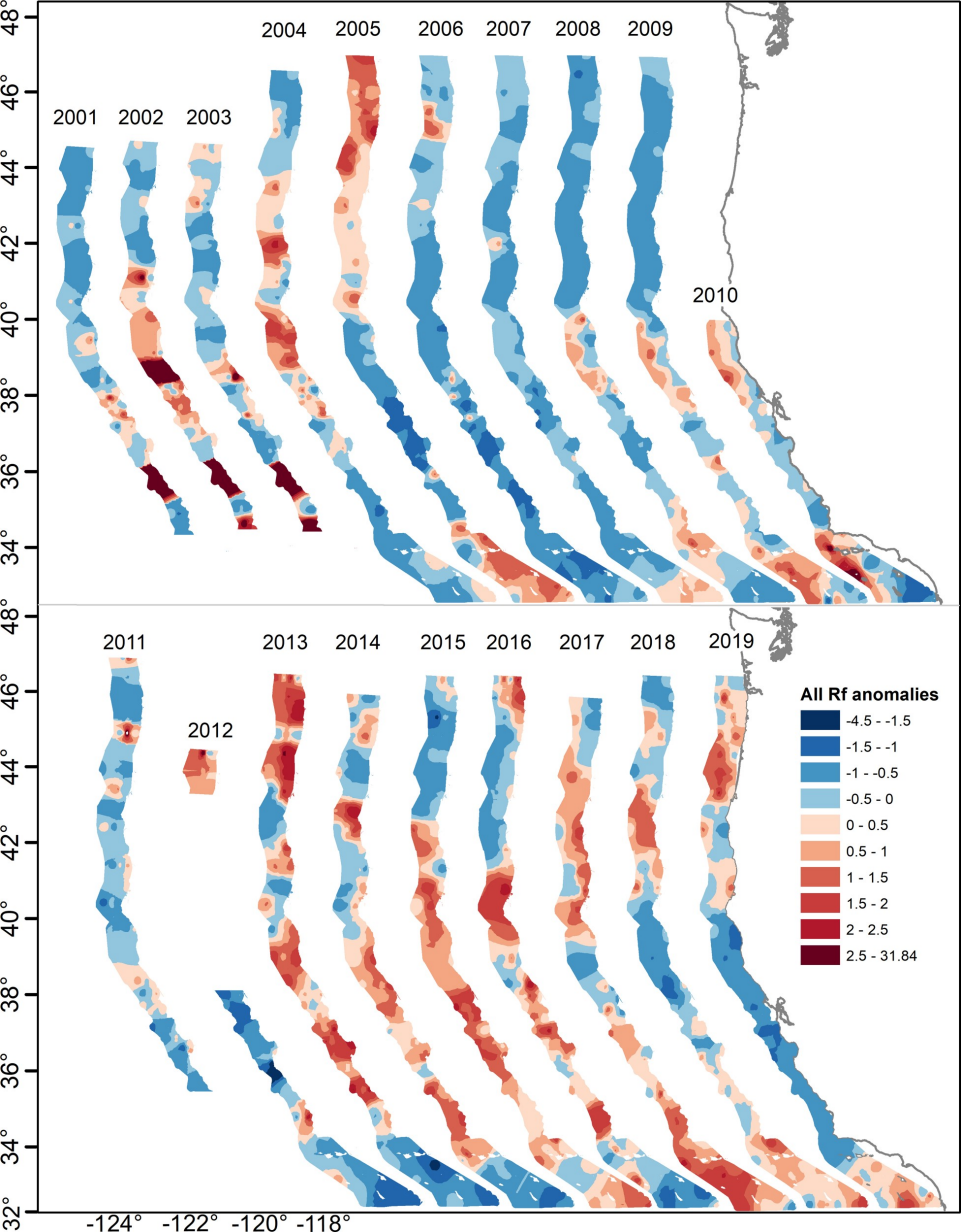
Interannual variability in relative pelagic young-of-year rockfish abundance, based on spatially explicit (station specific) z-scores from climatology years.

The average number of rockfish per haul ranged widely among years, from a low of 2.5 in 2006 to a high of 423 in 2015; well over two orders of magnitude (S1 Table). In particular, there were extremely high catch rates during 2013–2017, far higher than the early 2000s, which also represented a significant rise in catch rates from lows throughout the 1990s when the longer (1983–2019) time period is considered. These patterns are illustrated in the standardized anomalies of the resulting spatial climatology (Fig 4). In general, there was a tendency for spatial synchrony in the broad-scale abundance patterns during years of low or high abundance. For example, in addition to being very low in the central California region during the 2005–2008 time period, catch rates were generally low throughout the entire coastwide survey area from 2006 through 2007, and began to increase in the central and southern areas beginning in 2008 and 2009. Catches were very high in most areas between 2013 and 2017. However, a number of years demonstrated asynchronous regional patterns, such as in 2005, 2018, and 2019, when catch rates were high north of Cape Mendocino and south of Point Conception, while being at very low levels in the central region.

**Fig 5:**
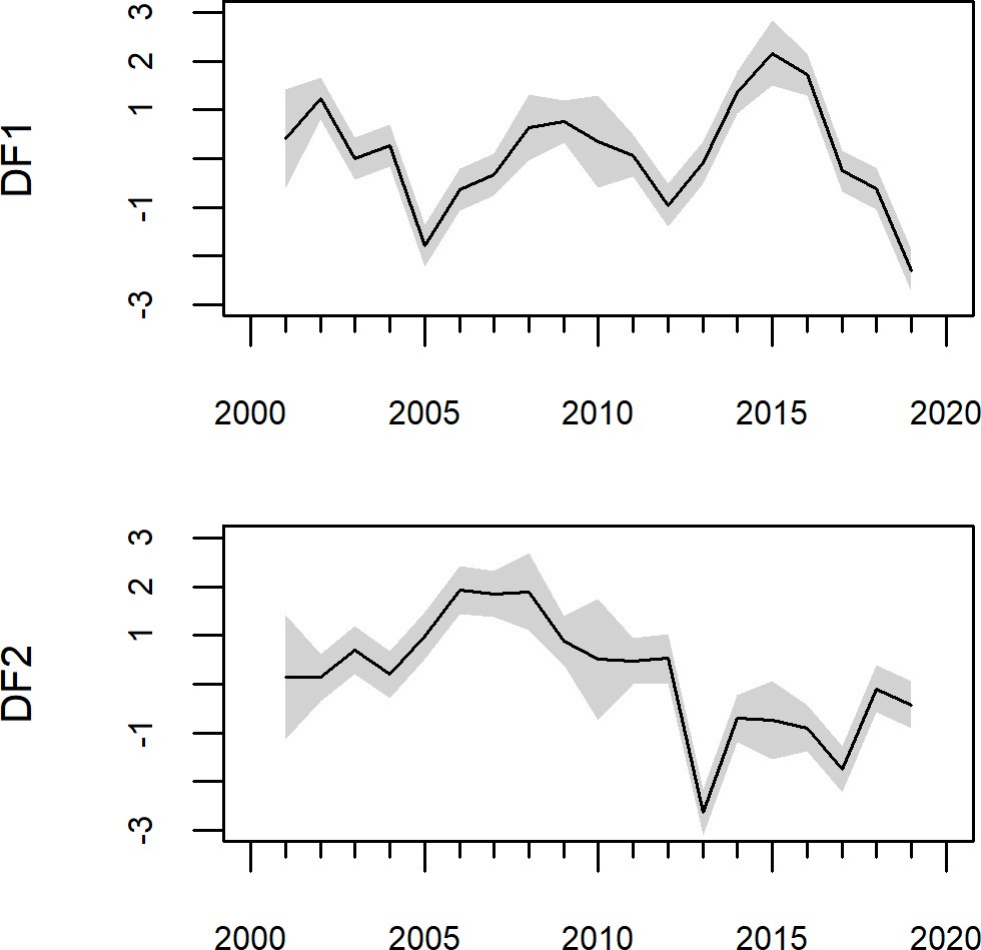
Trends (dynamic factors, or DFs) from the dynamic factor analysis though the 2001–2019 time period. Grey envelopes are 95% confidence intervals.

**Fig 6:**
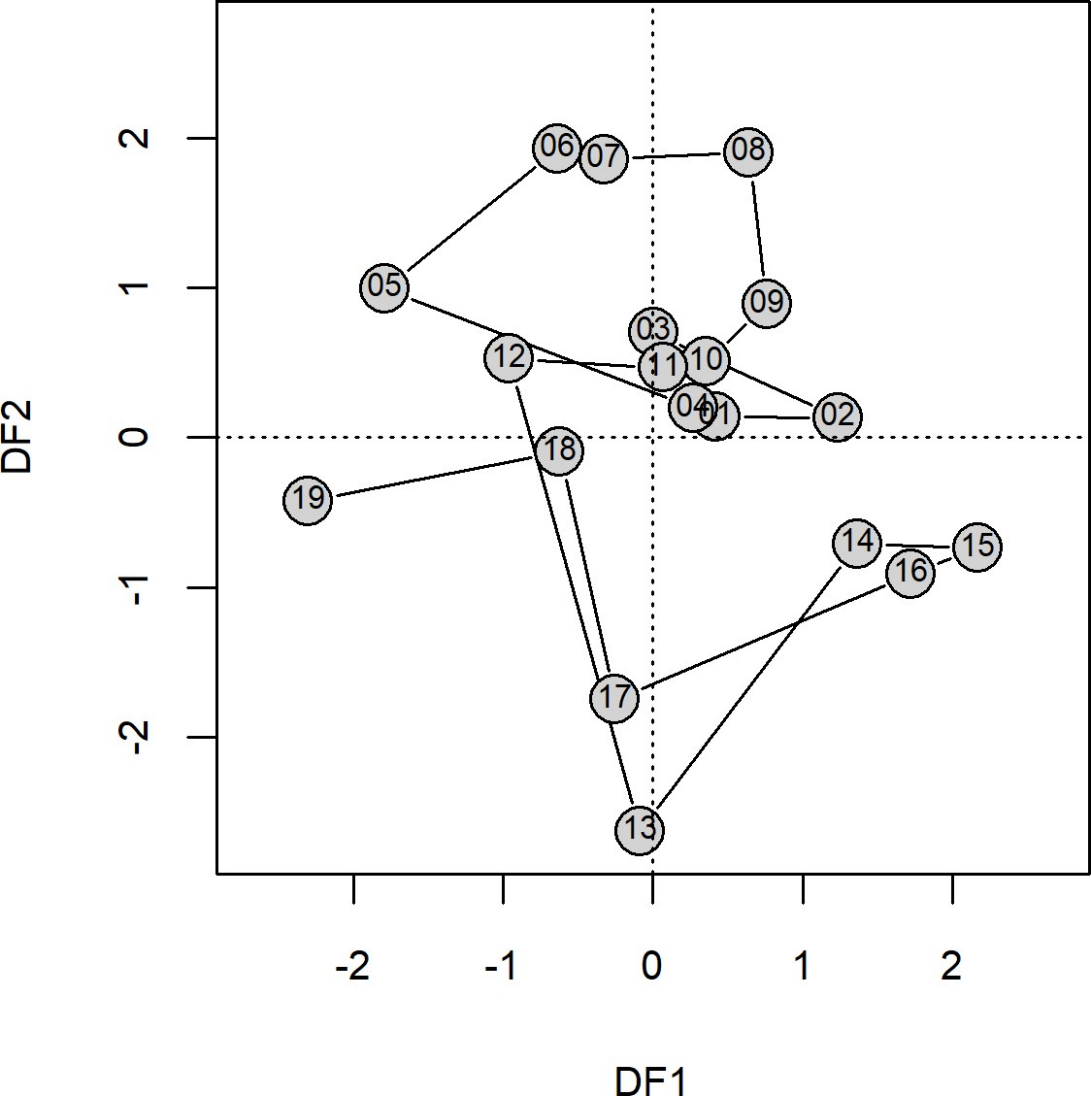
Biplot of Dynamic Factors (DFs) 1 and 2. Numbers within the points are the tens digit of year for 2001–2019 time series.

When regional trends were evaluated using DFA, the resulting model comparison found one model with a delta AICc less than 2.0 (S2 Table) and a weight of 0.574; thus we chose this model as the best-fitting model. This model had a two trends (dynamic factors, DF), a diagonal and unequal R with a single *ϕ* = 0.506 for all regions. Plotting these dynamic factors through time (Fig 5) shows that both trends show large changes around 2013, with DF1 increasing strongly and DF2 sharply decreasing for 2013. Both DFs correspond to the large increase of abundance of YOY rockfish observed coastwide during that time period. DF1 then began decreasing in 2015 while DF2 returned to more moderate values. A biplot of these trends (Fig 6) shows that the system state in terms of recruit abundance was very different in 2013–2017 relative to the rest of the time series, with the period from 2014–2016 being the most divergent. Given the ordination, 2013 and 2017 appear to be transitional years between normal and extreme marine heatwave conditions.

**Fig 7:**
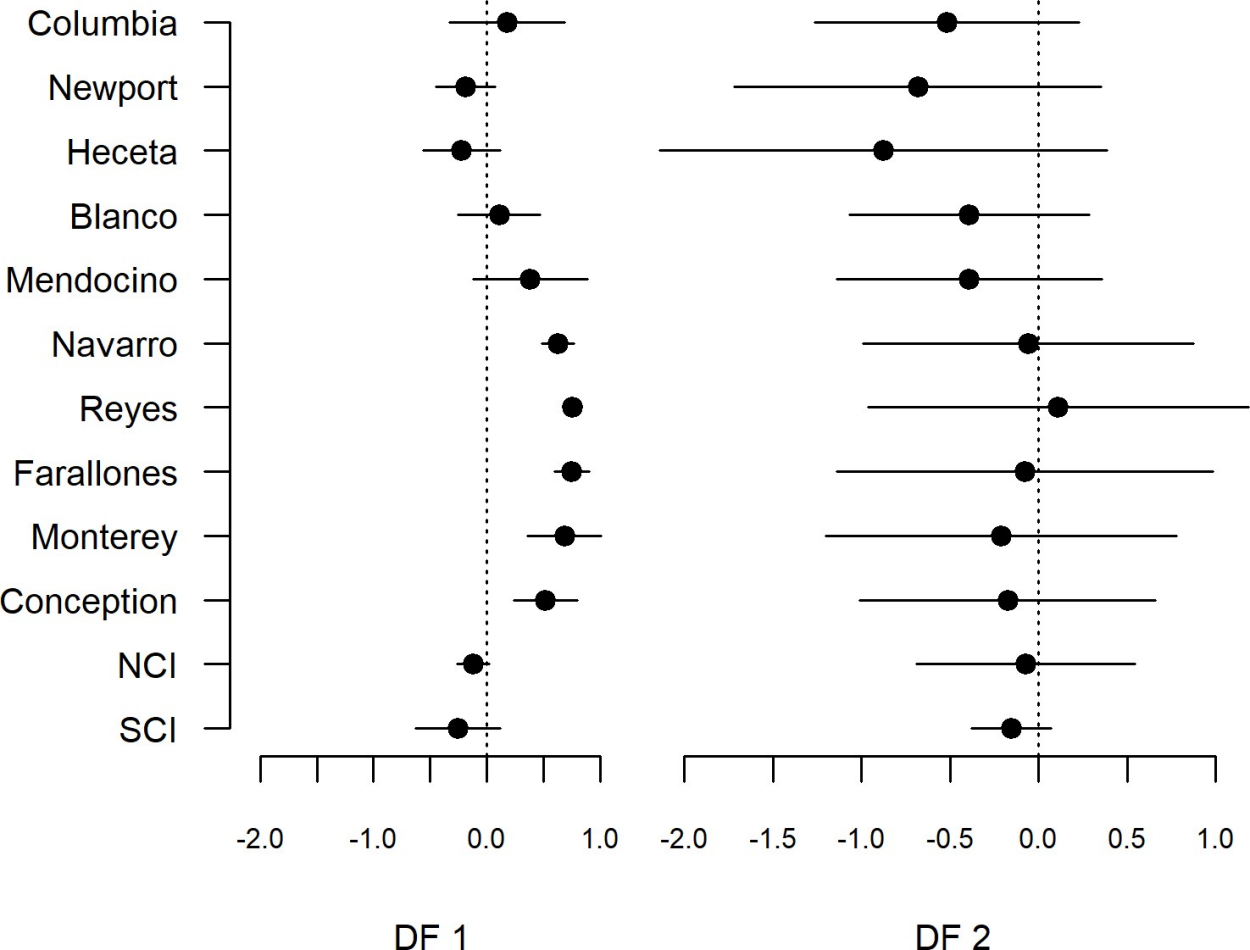
Loadings plot on regions from the dynamic factor analysis; regions are ordered north to south. Error bars are 95% confidence intervals. NCI and SCI are northern and southern Channel Islands, respectively. Note, the loading for SCI trend 2 has no error estimate because it was set to zero (pre-rotation) as a necessary requirement of constraining the DFA model.

The loadings on the two DFs reflect the degree of synchrony or asynchrony in the different regions of the California Current over time. Most of the central region, from the Conception area to the Blanco area, had strong positive loadings on DF1, as did the Columbia region (S3 Table, Fig 7), consistent with a considerable amount of shared covariation among regions in most years. However, the confidence intervals for several of these regions overlapped zero, indicating that the positive covariation was not definitive, and both the Newport and Heceta regions to the north, and the northern and southern Channel Islands regions in the south had negative loadings on this trend, indicating some regional variation in the abundance of pelagic recruits. For DF2, most regions had negative loadings, except for the Point Reyes region, again suggesting some level of coast-wide coherence in recruitment trends over space. However, loadings south of the Mendocino region were generally quite weak relative to those to the north. Collectively, the two factors seem to suggest that while a considerable fraction of regional variability is shared within years, the largest gradients in divergent trends tend to take place in the region between Capes Mendocino and Blanco.

**Fig 8:**
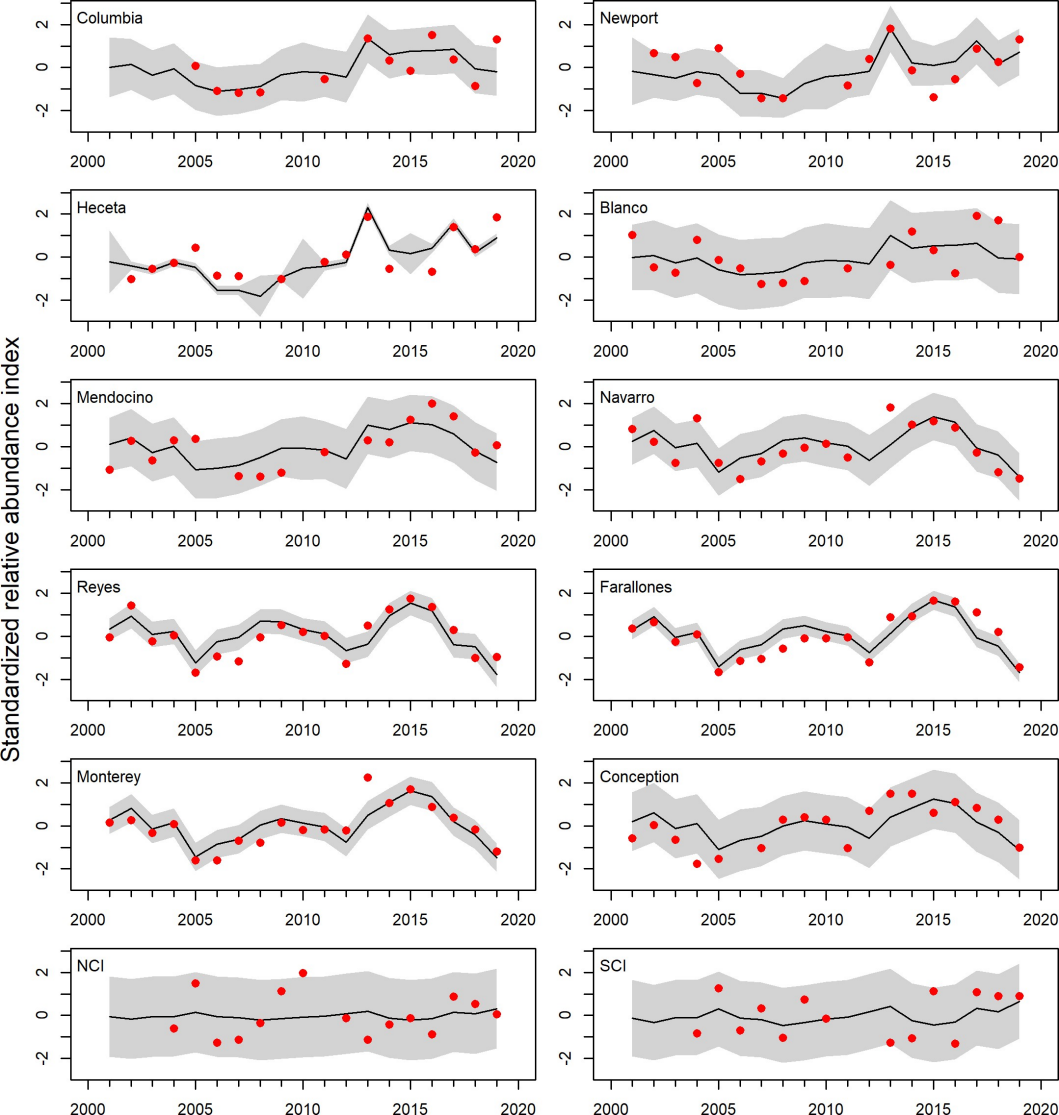
Fit of the two trend dynamic factor analysis to the regional catch rate data. Red dots are the observed estimates, the solid line is the dynamic factor analysis model estimate, and grey envelops are the 95% confidence intervals. Regions are ordered north to south.

Overall, the DFA model fit the observed time series well (Fig 8), with fits to the central regions (Navarro through Conception regions) consistent with the observed data, although there are clearly wider confidence intervals and poorer fits to the more sparsely sampled regions. The model fit the northern regions (Mendocino to Columbia regions) less well, and the model fits to the two southern regions (NCI and SCI areas) were relatively poor. In models that added a third trend, the fits to the NCI and SCI regions improved considerably, although the AICc values were more than 18 points higher (see S1 Fig). Although DFA is not able to assign a fraction of the total variance explained to a given factor, a PCA using the same time series (but excluding the years with incomplete coastal coverage) produced very comparable common trends and loadings to the two factors produced from the DFA for the climatology years (2004–2009, 2013–2019). The PCA had eigenvalues (fraction of deviance explained) of 42% and 26% for components 1 and 2, respectively, each of which was highly correlated with factors 1 and 2 from the DFA (see S3 Table, S2–S4 Figs). Collectively, these results suggest that approximately three quarters of the variability in coastwide juvenile rockfish abundance during the pelagic stage can be explained by the patterns associated with two general coastwide trends that are coherent across very broad, but not necessarily coastwide, spatial scales. Specifically, there is a general lack of coastwide synchrony between the Southern California Bight and the California Current waters further north, with a mix of synchronous and asynchronous abundance north and south of Cape Mendocino. However, there is considerable synchrony in catch rate signals between Point Conception and Cape Mendocino, and from Cape Mendocino to Southwest Washington and the Columbia River region.

### Species assemblage patterns

**Fig 9:**
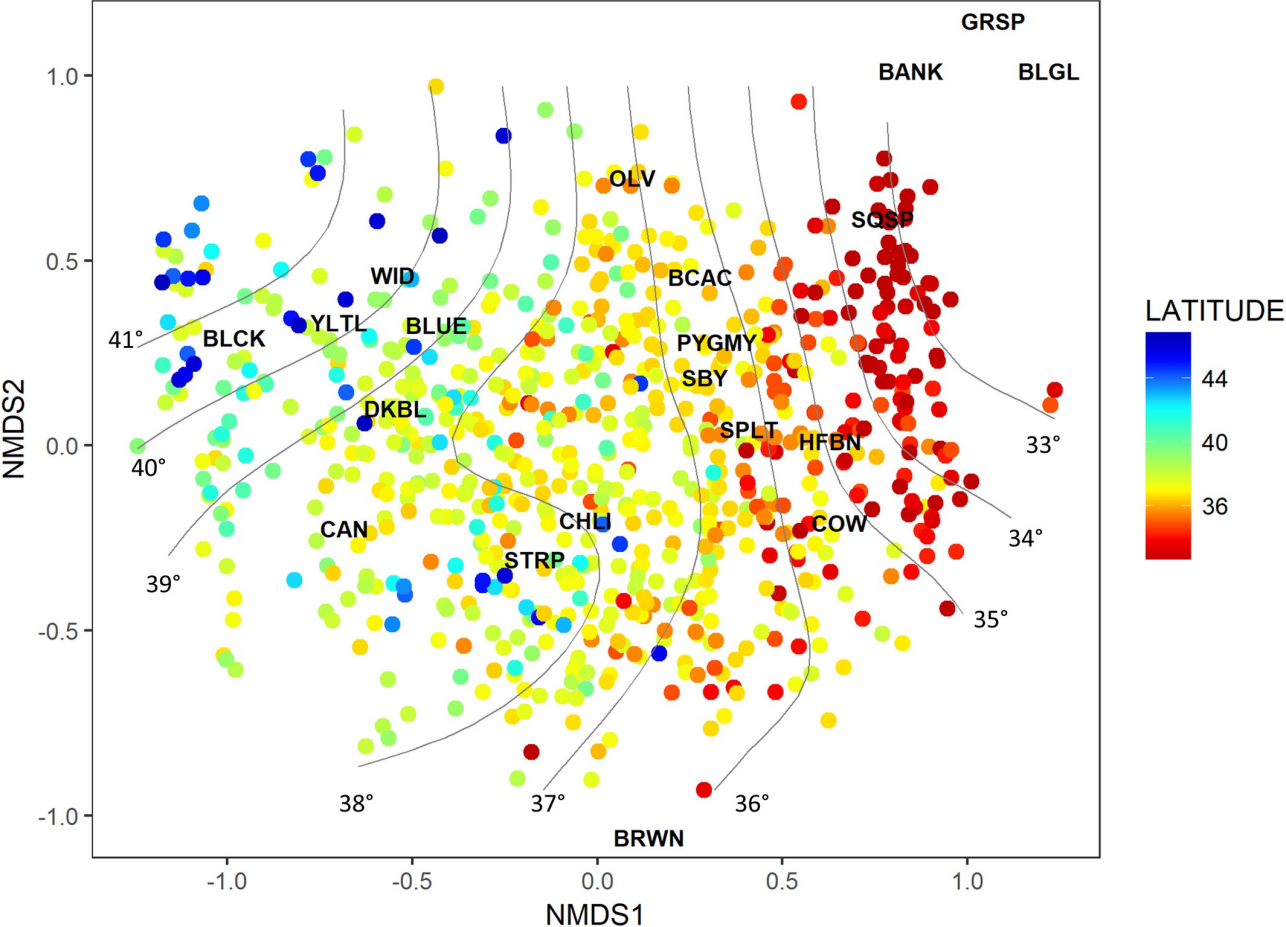
Spatial organization of rockfish taxa based on Non-metric Multi-Dimensional Scaling (NMDS) results. Latitude contours were predicted using a generalized additive model. Common names as follows, BLCK = Black, YLTL = Yellowtail, WID = Widow, BLUE = Blue/Deacon, DKBL = Darkblotched, CAN = Canary, STRP = Stripetail, CHIL = Chilipepper, BRWN = Brown, OLV = Olive, BCAC = Bocaccio, PYGMY = Pygmy, SBY = Shortbelly, SPLT = Splitnose, HFBN = Halfbanded, COW = Cowcod, SQSP = Squarespot, BANK = Bank, BLGL = Blackgill.

The NMDS analysis contributes further to our understanding of broad-scale latitudinal distribution and species association patterns (Fig 9). Overlaying latitude contours on the final ordination (3-dimensional NMDS, stress = 17.9) revealed several species clusters, with northern species such as black, blue, yellowtail, widow, darkblotched, and canary rockfishes clustering together at high- and mid-latitudes. However, it should be noted that the data in higher latitudes was sparse (only 60 hauls met the criteria of ≥ 3 species in the catch above 42^o^ N). Central and southern species such as chilipepper, bocaccio, and shortbelly rockfish tended to be found across a fairly wide range, but primarily in middle latitudes (36-40^o^ N), as did several species that tended to be more abundant in Southern California waters such as cowcod, splitnose, and halfbanded rockfish. A cluster of southern species such as squarespot, bank, and blackgill rockfishes was found almost exclusively in waters south of 36^o^ N.

## Discussion

The decision to expand the rockfish recruitment survey from central California to the entire U.S. West Coast was driven by the need to better understand larger-scale variability in catch rates, and to provide insights into just how much coastwide survey data was necessary to accurately estimate temporal variability in rockfish recruitment for commercially important species. This was triggered, in part, by the failure of the survey to observe the 1999 recruitment event, in which nearly every assessed rockfish stock in the California Current (and most other groundfish) showed strong to extremely strong recruitment [4, 5, 54]. The failure to observe this year-class in the central California survey area can be partially explained by extremely high upwelling conditions experienced during the 1999 survey that caused most juvenile rockfish to be advected far offshore of this core area while the survey was being conducted [55]. Although we cannot be certain that our survey would have recognized the strength of this year class had the spatial footprint been broader, the observation that high numbers of YOY were observed in power plan impingement and submersible surveys in southern California [15, 56] and in scuba surveys in northern California [30] for this year suggests that this would have been the case. Regardless, our results confirm that spatial and temporal patchiness are clearly a challenge to successful evaluation and enumeration of pre-recruits and subsequent development of effective pre-recruit indices for stock assessments, as has been previously observed in other comparable survey programs [20, 21].

One of the strong points of our sampling is the seasonal consistency in sampling over the time series of observations, which allowed us to make interannual comparisons of the catches over broad spatial scales. Sampling in the southern area generally occurred from early May through mid-June, while in the northern area it ranged from late May to early July. The timing of rockfish recruitment is very seasonal, particularly for the more northerly winter spawning species, and the survey was originally designed to include the time of peak abundance levels of these species in Central California [14]. More recently, based on monthly trawl sampling with a different gear type [57] confirmed that in the waters off of Oregon and Washington, the May-June period was most important for the larger commercially important species (e.g., widow, canary, and yellowtail rockfishes) while the later summer was characterized by higher catches of forage species. Despite this general finding, interannual differences in parturition times and growth rates could shift the availability of some species within this sampling window [58, 59]. Moreover, in southern waters, particularly in the southern California Bight, many rockfish species produce multiple broods, and spawning therefore may be spread across as many as 4 to 5 months, leading to greater opportunities to “miss” strong year classes in space and time [60–62]. Such processes could be factors with respect to the lack of consistency in the pre-recruit signal in the regions north and south of Point Conception. Greater investigations into the role of temporal variability in abundance, and the informative value of data collected during different time periods, would benefit future survey efforts.

While the combination of different time periods and provisioning strategies by species and region, as well as in response to demographic structure, all contribute to spatial and temporal variability, physical factors are well acknowledged to be key drivers of abundance and distribution patterns observed in these (and other) surveys. The timing and intensity of upwelling events is linked to both the growth and survival of larval and early juvenile stages, as well as the transport and advection or retention of the same from spawning grounds. Larval stages of rockfish and other groundfish are often associated cooler and less saline water masses, and greater larval abundance tends to be associated with years of greater southward flow in the California Current [63, 64]. However, years with strong upwelling can lead to advection of those larvae or later juvenile stages offshore, while years with dramatically reduced or delayed upwelling (for example 2005) are associated with lower productivity, leading to low survival and abundance, and potentially more inshore distributions of YOY, which may be less available to the trawl survey [65, 66]. These mesoscale features, dynamics, and patterns are contributing factors to the patchiness and variability observed regionally and interannually in these surveys, and indeed such patchiness and variability is now well known to be typical of the epipelagic macrozooplankton community more generally [23, 29, 67]. Despite this, large-scale physical factors are fairly well understood to drive overall abundance patterns and productivity of pelagic juvenile rockfish and the forage community more generally [11, 67, 68]. Such factors have also been associated with the abundance of recently settled juvenile rockfish [30] and patterns of recruitment variability estimated in stock assessment models for rockfish and other groundfish [54, 69, 70]. Most of those studies identified relative sea level as among the most explanatory environmental indicators for interpreting variable recruitment strength and community shifts, consistent with long-held observations that the strength of southward transport in the California Current is a key indicator of ecosystem productivity [4, 35, 71]. More recently, there is evidence that the mechanism is more tightly related to the origins of source waters at depth that are key drivers of variable YOY rockfish abundance, indicating that subsurface environmental conditions and processes are likely to be more important than surface conditions with respect to driving recruitment of commercially and ecologically important populations [11]. Specifically, the high fraction of Pacific Subarctic Upper Waters as source waters in the California Current during the large marine heatwave were consistent with the high abundance of YOY rockfish, despite the unusually warm surface waters and relatively high sea level observed during the 2014–2016 time period.

Moreover, the large-scale patterns described here appear to be associated with major biogeographic boundaries that similarly tend to define shifts in oceanographic provinces and species distributions [72, 73], including those for adult rockfishes [74, 75]. Although few studies have evaluated regional differences in recruitment patterns based on age-structured assessment models, one study that did so for three of the species included in this study, chilipepper, widow, and yellowtail rockfish, found large-scale patterns of covariability in year-class strength from central California to as far north as Canadian waters [76]. For these stocks, a common trend in recruitment explained 51% to 72% of the recruitment signal, depending upon the species, with the greatest differences among the regions were observed north and south of the Cape Mendocino region (none of the species examined included age and assessment data from south of Point Conception).

The observed pattern of greater YOY abundance throughout the southern extent of the coastwide region is somewhat surprising given that the abundance and catches of rockfish are generally thought to be significantly greater at higher latitudes. This appears to be largely driven by the relative abundance of the typically more diminutive forage taxa relative to fishery target taxa, with catches of the former centered in southern and central California waters, and the latter somewhat more evenly distributed throughout the California Current. Observations of larval rockfish community structure in the Southern California Bight are consistent with this finding, as over 80% of larvae were from forage species such as shortbelly, squarespot, and halfbanded rockfish, while less than 15% were from commercially important taxa such as bocaccio, bank, and widow rockfish [63]. The importance of both non-target and commercial taxa to predators and food webs is also substantial. Juvenile rockfish were the most frequently noted taxonomic group in a meta-analysis of food habits studies of top predators in the California Current [3], and prey switching by higher trophic level predators during periods of high or low abundance can have important cascading effects to both food webs and to fisheries [77, 78]. Thus, advection and other physical forcing processes have important implications not only for the spatial distribution of recruitment, but for regional food webs and top-down processes.

These results will help to inform sampling strategies for future applications of pre-recruit indices in stock assessments, particularly the observation of broad patterns in covariation over large spatial scales, but significant differences at the “biogeographic boundary” level spatial scales in this ecosystem (e.g., north of Cape Mendocino, south of Point Conception). This indicates that some data from all of these areas will likely to be necessary in order to develop robust recruitment indices, although smaller gaps in spatial coverage within these large-scale regions are likely to be acceptable, given the general coherence of trends within these broad biogeographic regions. However, as noted in the results, several years are characterized by catches in the historical core survey area that diverge substantially from those observed in the northern and southern regions of the survey area, particularly in 2005 when catches were among the lowest ever in the central California region, but were high north of Cape Mendocino and south of Point Conception. The unusual wind forcing that led to reduced upwelling and low primary and secondary productivity throughout the central part of California Current appear to have been described elsewhere [25, 28, 65]. Future efforts will more rigorously evaluate regional climate forcing as a driver of the abundance and distribution of YOY rockfish and other forage taxa, comparable to [11, 28, 29], but such efforts are beyond the scope of this analysis. Similarly, the extent to which the coastwide indices may perform better than those derived from a smaller regional scale, or whether some regions prove more informative with respect to incoming year class strength than others, continues to be under investigation. A key challenge in doing so is that confirmation of strong year classes within a stock assessment typically requires several years of data, and stock assessments for most of these populations are typically developed only once every 4 to 8 years. Thus, the current stock assessment estimates do not yet provide time series of sufficient duration to evaluate whether YOY abundance from some regions of the California Current “outperform” those from others with respect to predicting incoming year class strength. Despite this constraint and the associated time lags, recent stock assessments have confirmed some of the strong year classes suggested in DF1 from this analysis, including 2010, 2013, and 2014 for bocaccio, chilipepper, and widow rockfish [37, 38, 41], and 2013 for blue/deacon and darkblotched rockfish [39, 79]. Future studies will more rigorously evaluate how well the coastal indices perform relative to cohort strength based on fishery and survey demographic data, and in particular if the regional variability in abundance relates in any way to ultimate year class strength.

Other studies have shown that pre-recruit indices can provide robust information for informing future recruitment and cohort strength, even when such indices are based on surveys that do not completely overlap the spatial extent of the spawning stock [20, 21]. These studies have also found that forecasting tends to be more robust with later life-history stages (e.g., pelagic juvenile indices tend to outperform egg or larval indices), and that indices may be enhanced by combining empirical abundance data with environmental data [20]. Such an approach could be consistent for California Current rockfish given the consistent (albeit somewhat noisy) response of both pelagic juvenile and realized adult recruitment to large-scale advection and source water patterns in the California Current [4, 11, 54]. The shared variability among rockfish species observed in these studies also suggests that multispecies recruitment indices should be feasible, comparable to past efforts to provide shared indices of cohort strength for models for which demographic data are too sparse to provide clear signals at the single species level [5]. The results of the NMDS species assemblage patterns provide the basis for considering which taxa to pool into such multispecies indices, based on which species tend to covary in their spatial and temporal abundance patterns. Specifically, those results indicate that groupings of northern (including yellowtail, widow, black, blue, and darkblotched rockfish), central (chilipepper and stripetail) and southern (bocaccio, shortbelly, olive, and cowcod) may be candidates for multispecies recruitment indicators to inform future stock assessments. Simulation studies and potentially management strategy evaluations would benefit from a more rigorous consideration of the benefits and potential risks of improving year class strength estimates in rockfish (and other groundfish) stock assessment models, whether through pre-recruit surveys, environmental indicators, or some combination of the two [80]. While general benefits may be nominal under some circumstances, there are numerous examples in which management challenges arose from an unexpected high abundance, and consequent high catches, of smaller, younger fishes that were not anticipated in stock assessment models or other survey indices [15, 81].

In addition to informing assessment models, these surveys have provided a wide range of insights into early life history dynamics, which can be critical mechanisms to understand for long-term management needs, given the extent to which environment appears to drive most stock recruitment trends [6, 82]. Such knowledge is particularly important in light of the increasing use of ecosystem information to inform both stock assessments and fisheries managers [83, 84], as well as the expectation that marine ecosystem processes and dynamics will vary substantially in the face of future climate change. Finally, such surveys also provide key insights into other components of the food web, providing helpful food web and ecosystem insights, such as the consequences of prey switching by predators to other components of the food web, that can also prove informative to management [19, 78, 85]. As such, the products of these surveys will benefit marine resource management and stewardship at multiple levels.

## Supporting information

S1 Appendix Raw haul and catch data used to inform the analyses, including survey, year, date, latitude, longitude, station, and region of each haul, as well as catch of YOY rockfish at the lowest taxonomic resolution available. (CSV)

S2 Appendix (XLSX)

S1 Table Scientific names, common names, and associated catch and life history information for *Sebastes* encountered in the coastwide survey, 2001–2019. Information includes total numbers encountered, fraction of total catch, designation as target or ecosystem taxa, estimated L_infinity_ value and source for each value.
(DOCX)

S2 Table Model selection statistics: Dynamic factor analysis AICcs and model weights. (DOCX)

S3 Table Dynamic factor regional loadings, including upper and lower 95% confidence intervals. (DOCX)

S4 Table Component loadings for the principal components analysis. (DOCX)

S1 Fig Fit of the three-trend DFA model to the observed data. The model had three-trends and diagonal and equal R matrix. Red points are observed data. Solid line is the model fit and grey envelopes are the ~95% confidence limits.
(TIF)

S2 Fig Scree plot showing variance explained by each PC axis. PC’s 1 and 2 explain 42 and 26% of the variance in the 2004–2009, 2013–2019 time series, respectively.
(TIF)

S3 Fig The first three principal coordinates from the PCA analysis plotted through time. (TIF)

S4 Fig Correlations between the first three principal components and Dynamic Factors (DFs) 1 and 2 from the best fitting Dynamic Factor Analysis model. (TIF)
