# Supplementary material for: Spatiotemporal patterns of variability in the abundance and distribution of winter-spawned pelagic juvenile rockfish in the California Current
Source: PLoS One. 2021 May 27;16(5):e0251638. doi: 10.1371/journal.pone.0251638 (PMC8158922; doi:10.1371/journal.pone.0251638)
Supplement: S1 Table — Information includes total numbers encountered, fraction of total catch, designation as target or ecosystem taxa, estimated Linfinity value and source for each value. (DOCX) [file pone.0251638.s003.docx]

S1 Table: Scientific names, common names, and associated catch and life history information for Sebastes encountered in the coastwide survey, 2001-2019.

| Scientific name | Common name | total catch | % catch | group | L_inf_ | L_inf_ source |
| --- | --- | --- | --- | --- | --- | --- |
| *S. auriculatus* | Brown | 1581 | 0.5% | target | 51.4 | [1] |
| *S. aurora* | Aurora | 7 | <0.1% | target | 31.4 | [2] |
| *S. babcocki* | Redbanded | 6 | <0.1% | target | N/A | N/A |
| *S. caurinus, S. carnatus** | Copper Group | 1778 | 0.6% | target | N/A | N/A |
| *S. crameri* | Darkblotched | 439 | 0.1% | target | 43 | [3] |
| *S. dallii* | Calico | 275 | 0.1% | forage | 16.2 | [4] |
| *S. diploproa* | Splitnose | 66 | <0.1% | target | 29.6 | [5] |
| *S. elongatus* | Greenstripe | 507 | 0.1% | target | 33.8 | [6] |
| *S. emphaeus* | Puget Sound | 1 | <0.1% | forage | 17.1 | [7] |
| *S. entomelas* | Widow | 16929 | 6.2% | target | 50.7 | [8] |
| *S. flavidus* | Yellowtail | 3439 | 1.2% | target | 55.2 | [9] |
| *S. goodei* | Chilipepper | 15399 | 5.6% | target | 48.1 | [10] |
| *S. hopkinsi* | Squarespot | 12190 | 4.4% | forage | 25.3 | [4] |
| *S. jordani* | Shortbelly | 169913 | 62.3% | forage | 28.5 | [11] |
| *S. levis* | Cowcod | 447 | 0.1% | target | 73.9 | [12] |
| *S. melanops* | Black | 633 | 0.2% | target | 55 | [13] |
| *S. melanostomus* | Blackgill | 549 | 0.2% | target | 63.5 | [14] |
| *S. miniatus/S. crocotulus* | Vermillion/Sunset | 47 | <0.1% | target | 53.7 | [15] |
| *S. mystinus/S. diaconus*** | Blue/Deacon | 5397 | 1.9% | target | 38.1 | [16] |
| *S. ovalis* | Speckled | 1 | <0.1% | target | 50 | [4] |
| *S. paucispinis* | Bocaccio | 1426 | 0.5% | target | 67.5 | [17] |
| *S. pinneger* | Canary | 2173 | 0.7% | target | 61.3 | [18] |
| *S. rastrelliger* | Grass | 5 | <0.1% | target | 51.3 | [19] |
| *S. reedi* | Yellowmouth | 102 | <0.1% | target | 46.4 | [4] |
| *S. ruberrimus* | Yelloweye | 39 | <0.1% | target | 64.1 | [20] |
| *S. rufus* | Bank | 503 | 0.1% | target | 59.4 | [21] |
| *S. saxicola* | Stripetail | 9919 | 3.6% | forage | 33 | [4] |
| *S. semicinctus* | Halfbanded | 17110 | 6.2% | forage | 18.1 | [4] |
| *S. serranoides* | Olive | 222 | <0.1% | target | 51.9 | [22] |
| *S. serriceps* | Treefish | 3 | <0.1% | target | 30.6 | [23] |
| *S. wilsoni* | Pygmy | 838 | 0.3% | forage | N/A | N/A |
| *S. zacentrus* | Sharpchin | 43 | <0.1% | target | 35 | [24] |
| *Sebastes spp.* | Unidentified | 2753 | 1.0% | n/a | N/A | N/A |
| *Sebastomus spp.**** | Rosy group | 7620 | 2.7% | target | N/A | N/A |

* The "Copper group" includes Copper (*S. caurinus*), Gopher (*S. carnatus*), Black and Yellow (*S. chrysomelas*) and Kelp (*S. atrovirens*), which are difficult to differentiate to the species level.

** Blue and Deacon rockfishes were described as separate species [25], and pelagic juveniles cannot yet be morphologically identified to the species level. However distribution patterns of YOY by species have been described based on a subset of pelagic YOY that were identified using molecular methods [26].

*** The "Rosy" group include species in the sub-genera Sebastomus not otherwise identified to the species level, such as Rosy (*S. rosaceus*), Greenspotted (*S. chlorostictus*), Swordspine (*S. ensifer*) and starry (*S. constellatus*) rockfish.

Sources for S.1

1. Cope J, Dick EJ, MacCall AD, Monk MM, Soper B, Wetzel C. Data-moderate stock assessments for brown, China, copper, sharpchin, stripetail, and yellowtail rockfishes and English and rex soles in 2013. Pacific Fishery Management Council, Portland, OR. 2013.

2. Hamel OS, Cope JM, Matson S. Stock assessment of aurora rockfish in 2013. Pacific Fishery Management Council, Portland, OR. 2013.

3. Wallace DR, Gertseva V. Status of the darkblotched rockfish resource off the continental U.S. Pacific Coast in 2017 (Update of 2015 assessment model). Pacific Fishery Management Council, Portland, OR. 2018.

4. Love MS, Yoklavich M, Thorsteinson LK. The rockfishes of the northeast Pacific. University of California Press. 2002.

5. Gertseva V, Cope JM, Pearson D. Status of the U.S. splitnose rockfish (*Sebastes diploproa*) resource in 2009. Pacific Fishery Management Council, Portland, OR. 2009.

6. Hicks, A., Haltuch, M., C.R. Wetzel. Status of greenstriped rockfish (*Sebastes elongatus*) along the outer coast of California, Oregon, and Washington. Pacific Fishery Management Council, Portland, OR. 2009.

7. Beckmann AT, Gunderson DR, Miller BS, Buckley RM, Goetz B. Reproductive biology, growth, and natural mortality of Puget Sound rockfish, *Sebastes emphaeus* (Starks, 1911). Fishery Bulletin. 1998;96(2): 352-356.

8. Adams GD, Kapur MS, McQuaw K, Thurner S, Hamel OS, Stephens A, et al. Stock assessment update: status of widow rockfish (*Sebastes entomelas*) along the U.S. West Coast in 2019. Pacific Fishery Management Council, Portland, Oregon. 2019.

9. Stephens A, Taylor IG. Status of Yellowtail Rockfish (*Sebastes flavidus*) Along the U.S. Pacific Coast in 2017. Pacific Fishery Management Council, Portland, OR. 2018.

10. Field JC, Beyer S, He X. Status of the chilipepper rockfish, *Sebastes goodei*, in the California Current for 2015. Pacific Fishery Management Council, Portland, Oregon. 2017.

11. Field JC, Dick EJ, MacCall AD. Stock assessment model for the shortbelly rockfish, *Sebastes jordani*, in the California Current. NOAA Technical Memorandum NMFS/SWFSC 405. 83pp. 2007.

12. Dick EJ, He X. Status of Cowcod (*Sebastes levis*). Pacific Fishery Management Council. Portland, OR. 2019.

13. Cope JM, Sampson D, Stephens A, Key M, Mirick PP, Stachura M, et al. Assessments of California, Oregon and Washington stocks of black rockfish (*Sebastes melanops*) in 2015. Pacific Fishery Management Council. Portland, OR. 2015.

14. Field JC, He X.Stock assessment update of blackgill rockfish, *Sebastes melanostomus*, in the Conception and Monterey INPFC areas for 2017. Pacific Fishery Management Council. Portland, OR. 2018.

15. MacCall AD. Assessment of vermilion rockfish in southern and northern California. Pacific Fishery Management Council, Portland, OR. 2005.

16. Dick EJ, Berger A, Bizzarro J, Bosley K, Cope J, Field J, et al. The combined status of blue and deacon rockfishes in U.S. waters off California and Oregon in 2017. Pacific Fishery Management Council, Portland, Oregon. 2017.

17. He X, Field JC.. Stock assessment update: status of bocaccio, *Sebastes paucispinis*, in the Conception, Monterey and Eureka INPFC areas for 2017. Pacific Fishery Management Council. Portland, OR. 2017.

18. Thorson J, Wetzel C. The status of canary rockfish (*Sebastes pinniger*) in the California Current in 2015. Pacific Fishery Management Council. Portland, OR. 2015.

19. Love MS, Johnson K. Aspects of the life histories of the grass rockfish (*Sebastes rastrelliger*) and brown rockfish (*S. auriculatus*) from southern California. Fish Bull. 1999;97: 100-109.

20. Gertseva V, Cope JM. Stock assessment of the yelloweye rockfish (*Sebastes ruberrimus*) in state and federal waters off California, Oregon and Washington. Pacific Fishery Management Council, Portland, OR. 2017.

21. Watters DL, Kline DE, Coale KH, Cailliet GM. Radiometric age confirmation and growth of a deep-water marine fish species: the bank rockfish, *Sebastes rufus*. Fish Res. 2006;81: 251-257.

22. Love MS, Westphal W. Growth, reproduction, and food habits of olive rockfish, Sebastes serranoides, off central California. Fish Bull. 1981;79: 533-545.

23. Colton, MA, Larson RJ. Aspects of the life history of treefish, *Sebastes serriceps* (Sebastidae). CalCOFI Rep. 2007;48: 177-190.

24. Malecha PW, Hanselman DH, Heifetz J. Growth and mortality of rockfishes (Scorpaenidae) from Alaska waters. U.S. Dep. Commer, NOAA Tech. Memo. NMFS-AFSC-172, 2007.61 p.

25. Frable BW, Wagman DW, Frierson TN, Aguilar A, Sidlauskas BL. A new species of *Sebastes* (Scorpaeniformes: Sebastidae) from the northeastern Pacific, with a redescription of the blue rockfish, *S. mystinus* (Jordan and Gilbert, 1881). Fish Bull. 2015;113: 355-377.

26. Bizzarro JJ, Gilbert-Horvath EA, Dick EJ, Berger AM, Schmidt KT, Pearson D, Petersen C, Kautzi LA, Miller RR, Field JC, Garza JC. Genetic identification of blue rockfish (*Sebastes mystinus*) and deacon rockfish (*S. diaconus*) to enable life history analyses for stock assessment. Fish Bull. 2020; 118: 37-55.
