# Supplementary material for: Spatiotemporal patterns of variability in the abundance and distribution of winter-spawned pelagic juvenile rockfish in the California Current
Source: PLoS One. 2021 May 27;16(5):e0251638. doi: 10.1371/journal.pone.0251638 (PMC8158922; doi:10.1371/journal.pone.0251638)
Supplement: S2 Table — (DOCX) [file pone.0251638.s004.docx]

S2 Table: Model selection statistics: dynamic factor analysis AICcs and model weights.

| Model | AIC | AICc | delta_AICc | Weight |
| --- | --- | --- | --- | --- |
| Trends = 2 R = diagonal and unequal B = diagonal and equal | 485.9604 | 502.5070 | 0.000000 | 0.574 |
| Trends = 2 R = diagonal and unequal B = diagonal and unequal | 487.7675 | 505.3425 | 2.835529 | 0.139 |
| Trends = 2 R = diagonal and equal B = diagonal and equal | 498.6297 | 506.1879 | 3.680903 | 0.091 |
| Trends = 2 R = diagonal and equal B = diagonal and unequal | 499.0303 | 507.2408 | 4.733836 | 0.054 |
| Trends = 1 R = diagonal and unequal B = diagonal and equal | 501.0496 | 508.6077 | 6.100781 | 0.027 |
| Trends = 1 R = diagonal and unequal B = diagonal and unequal | 501.0496 | 508.6077 | 6.100781 | 0.027 |
| Trends = 2 R = diagonal and unequal B = identity | 493.1485 | 508.7041 | 6.197148 | 0.026 |
| Trends = 3 R = diagonal and unequal B = diagonal and equal | 480.2549 | 508.8906 | 6.383684 | 0.024 |
| Trends = 1 R = diagonal and unequal B = identity | 502.8818 | 509.8182 | 7.311273 | 0.015 |
| Trends = 2 R = diagonal and equal B = identity | 503.1934 | 510.1298 | 7.622818 | 0.013 |
| Trends = 3 R = diagonal and equal B = diagonal and equal | 496.1792 | 511.7347 | 9.227753 | 0.006 |
| Trends = 3 R = diagonal and equal B = diagonal and unequal | 496.0168 | 513.5918 | 11.084808 | 0.002 |
| Trends = 3 R = diagonal and unequal B = diagonal and unequal | 483.1921 | 514.7626 | 12.255622 | 0.001 |
| Trends = 1 R = diagonal and equal B = diagonal and equal | 515.3832 | 517.6783 | 15.171326 | 0.000 |
| Trends = 1 R = diagonal and equal B = diagonal and unequal | 515.3832 | 517.6783 | 15.171326 | 0.000 |
| Trends = 1 R = diagonal and equal B = identity | 516.2997 | 518.2780 | 15.771051 | 0.000 |
| Trends = 3 R = diagonal and unequal B = identity | 493.3560 | 520.5928 | 18.085872 | 0.000 |
| Trends = 3 R = diagonal and equal B = identity | 506.1657 | 520.7669 | 18.259984 | 0.000 |
