# Supplementary material for: Spatiotemporal patterns of variability in the abundance and distribution of winter-spawned pelagic juvenile rockfish in the California Current
Source: PLoS One. 2021 May 27;16(5):e0251638. doi: 10.1371/journal.pone.0251638 (PMC8158922; doi:10.1371/journal.pone.0251638)
Supplement: S3 Table — (DOCX) [file pone.0251638.s005.docx]

S3 Table: Dynamic Factor regional loadings, including upper and lower 95% confidence intervals.

| Region | Trend 1 | Trend 2 | CI_low1 | CI_up1 | CI_low2 | CI_up2 |
| --- | --- | --- | --- | --- | --- | --- |
| Columbia | 0.179 | -0.517 | -0.330 | 0.688 | -1.262 | 0.227 |
| Newport | -0.189 | -0.681 | -0.452 | 0.074 | -1.718 | 0.355 |
| Heceta | -0.224 | -0.875 | -0.565 | 0.117 | -2.138 | 0.387 |
| Blanco | 0.109 | -0.391 | -0.253 | 0.470 | -1.068 | 0.287 |
| Mendocino | 0.383 | -0.392 | -0.120 | 0.887 | -1.141 | 0.358 |
| Navarro | 0.625 | -0.059 | 0.483 | 0.768 | -0.993 | 0.876 |
| Reyes | 0.750 | 0.112 | 0.820 | 0.681 | -0.960 | 1.185 |
| Farallones | 0.749 | -0.075 | 0.595 | 0.903 | -1.138 | 0.987 |
| Monterey | 0.684 | -0.211 | 0.360 | 1.008 | -1.201 | 0.779 |
| Conception | 0.515 | -0.174 | 0.234 | 0.797 | -1.009 | 0.660 |
| NCI | -0.123 | -0.071 | -0.265 | 0.019 | -0.688 | 0.545 |
| SCI | -0.256 | -0.154 | -0.629 | 0.117 | -0.379 | 0.070 |
