# Supplementary material for: Spatiotemporal patterns of variability in the abundance and distribution of winter-spawned pelagic juvenile rockfish in the California Current
Source: PLoS One. 2021 May 27;16(5):e0251638. doi: 10.1371/journal.pone.0251638 (PMC8158922; doi:10.1371/journal.pone.0251638)
Supplement: S4 Table — (DOCX) [file pone.0251638.s006.docx]

S4 Table: Component loadings for the Principal Components Analysis.
