# Supplementary figures and images for: Spatiotemporal patterns of variability in the abundance and distribution of winter-spawned pelagic juvenile rockfish in the California Current
Source: PLoS One. 2021 May 27;16(5):e0251638. doi: 10.1371/journal.pone.0251638 (PMC8158922; doi:10.1371/journal.pone.0251638)

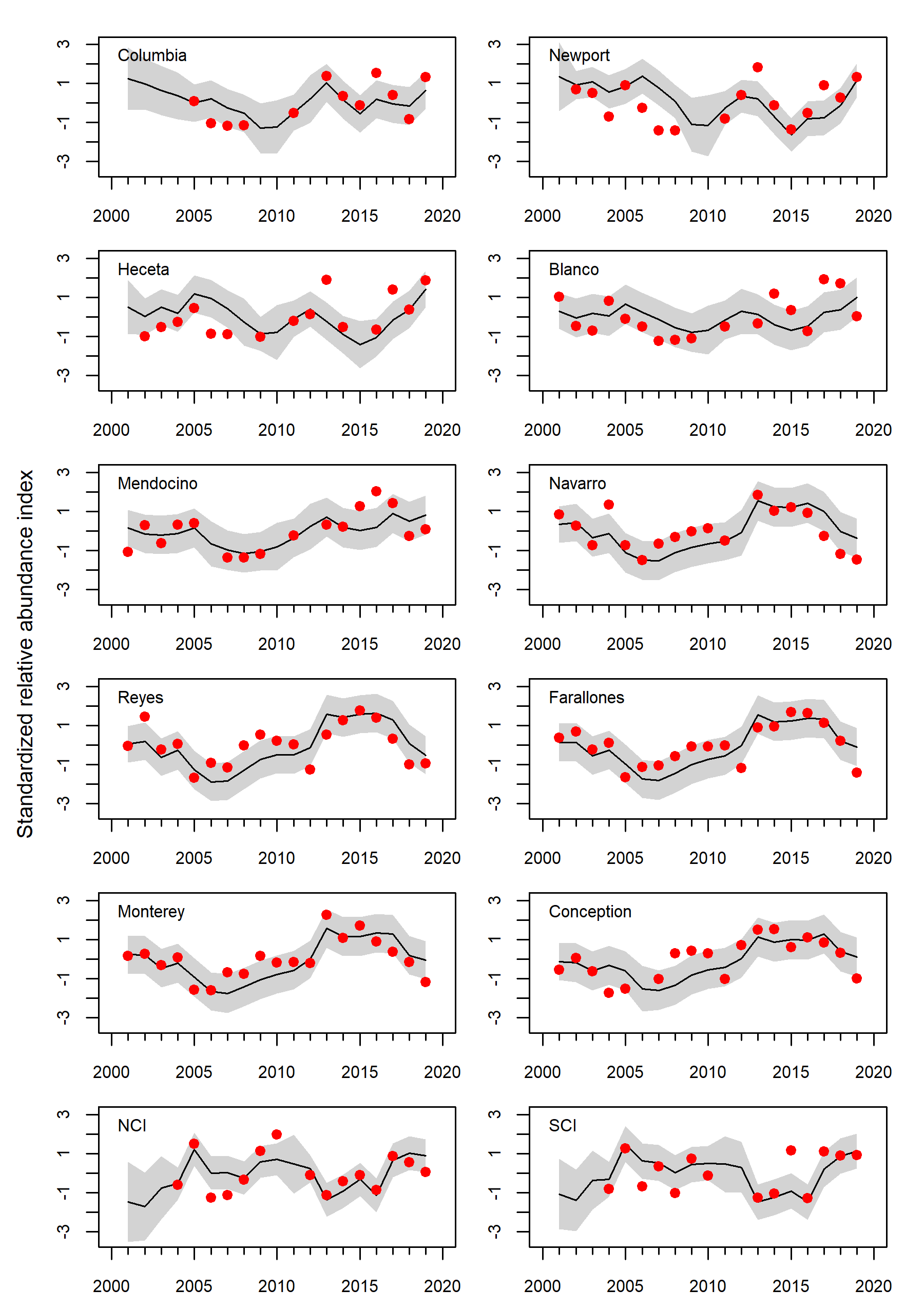

Supplement: S1 Fig — The model had three-trends and diagonal and equal R matrix. Red points are observed data. Solid line is the model fit and grey envelopes are the ~95% confidence limits. (TIF) [file pone.0251638.s007.tif]

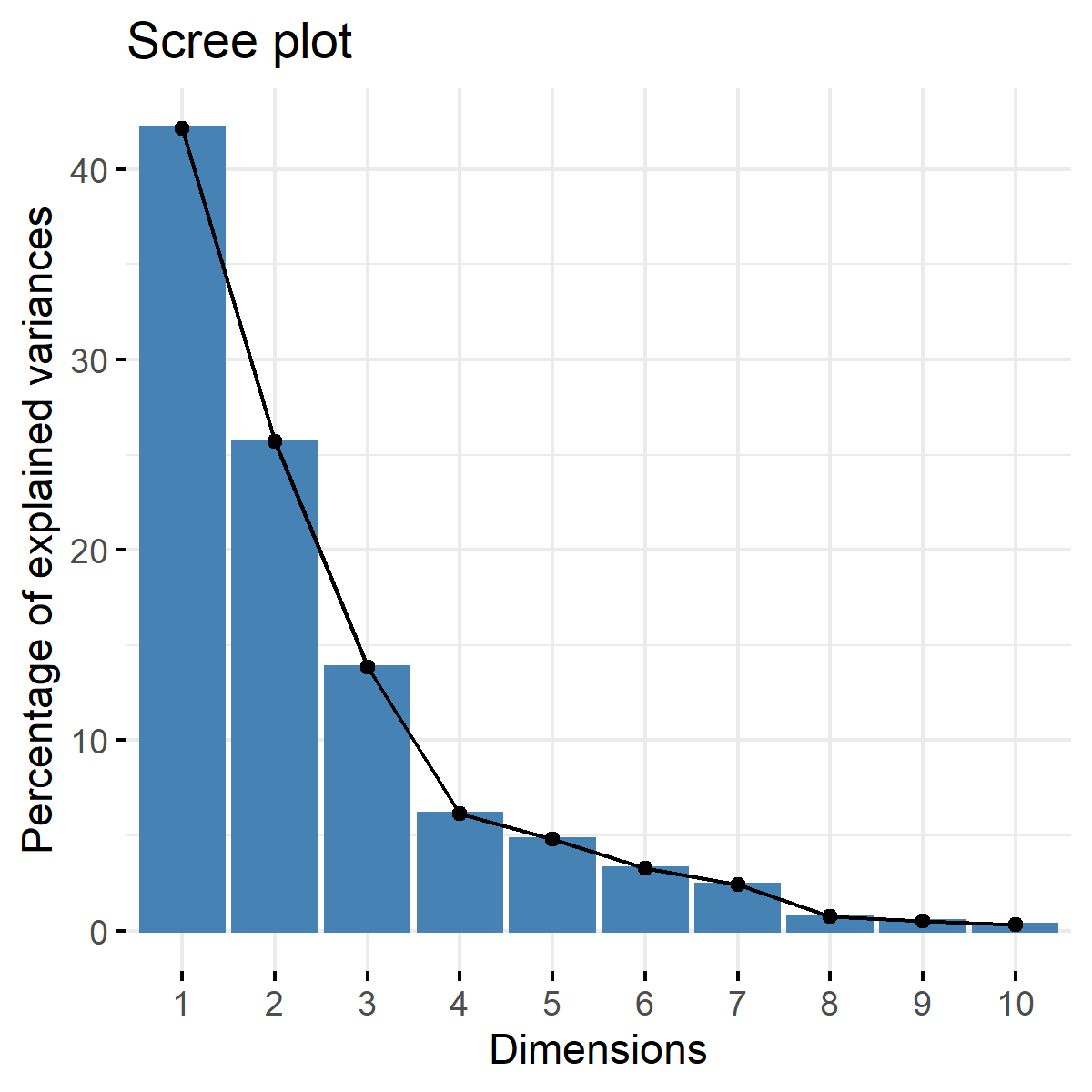

Supplement: S2 Fig — PC’s 1 and 2 explain 42 and 26% of the variance in the 2004–2009, 2013–2019 time series, respectively. (TIF) [file pone.0251638.s008.tif]

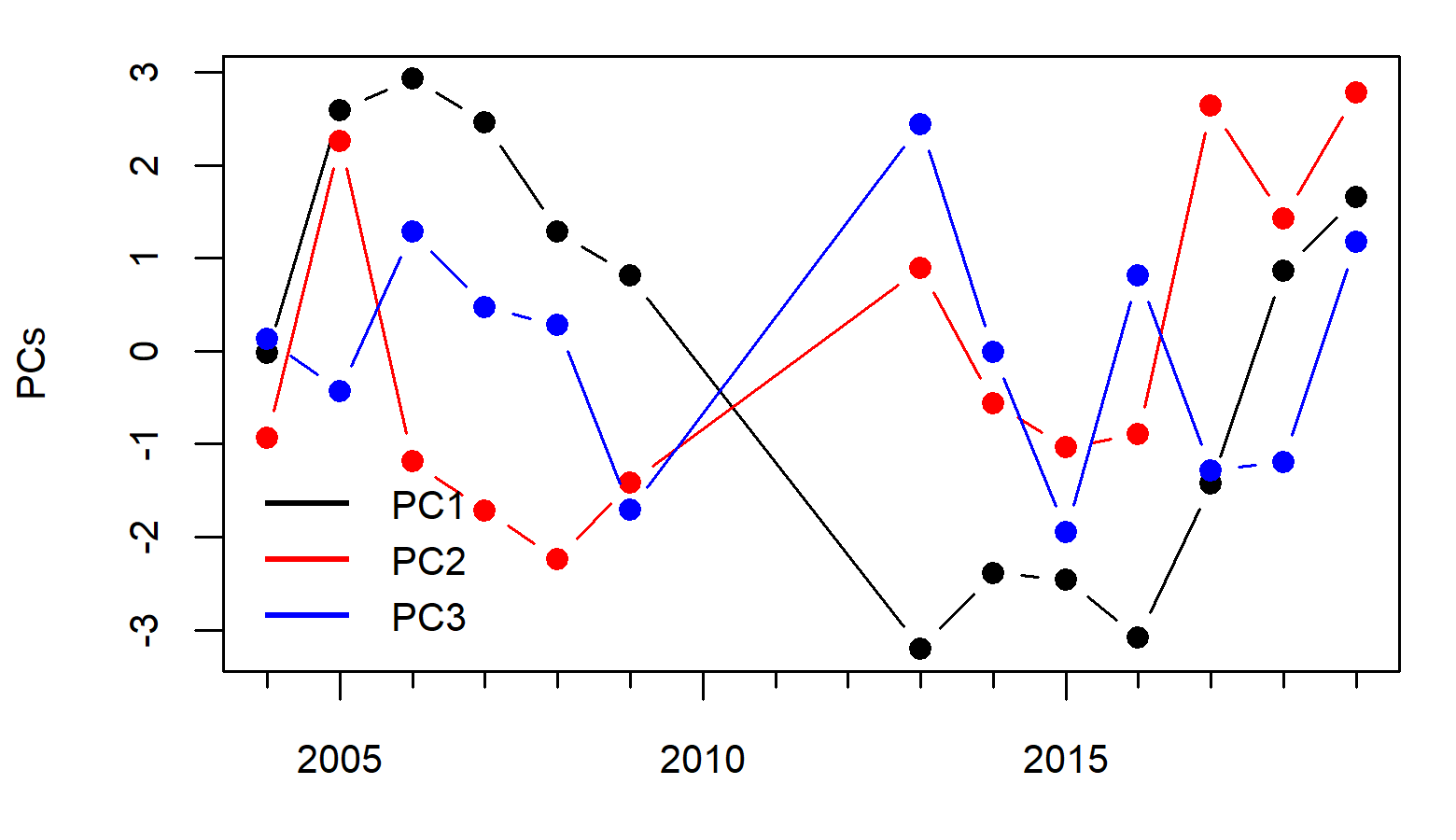

Supplement: S3 Fig — (TIF) [file pone.0251638.s009.tif]

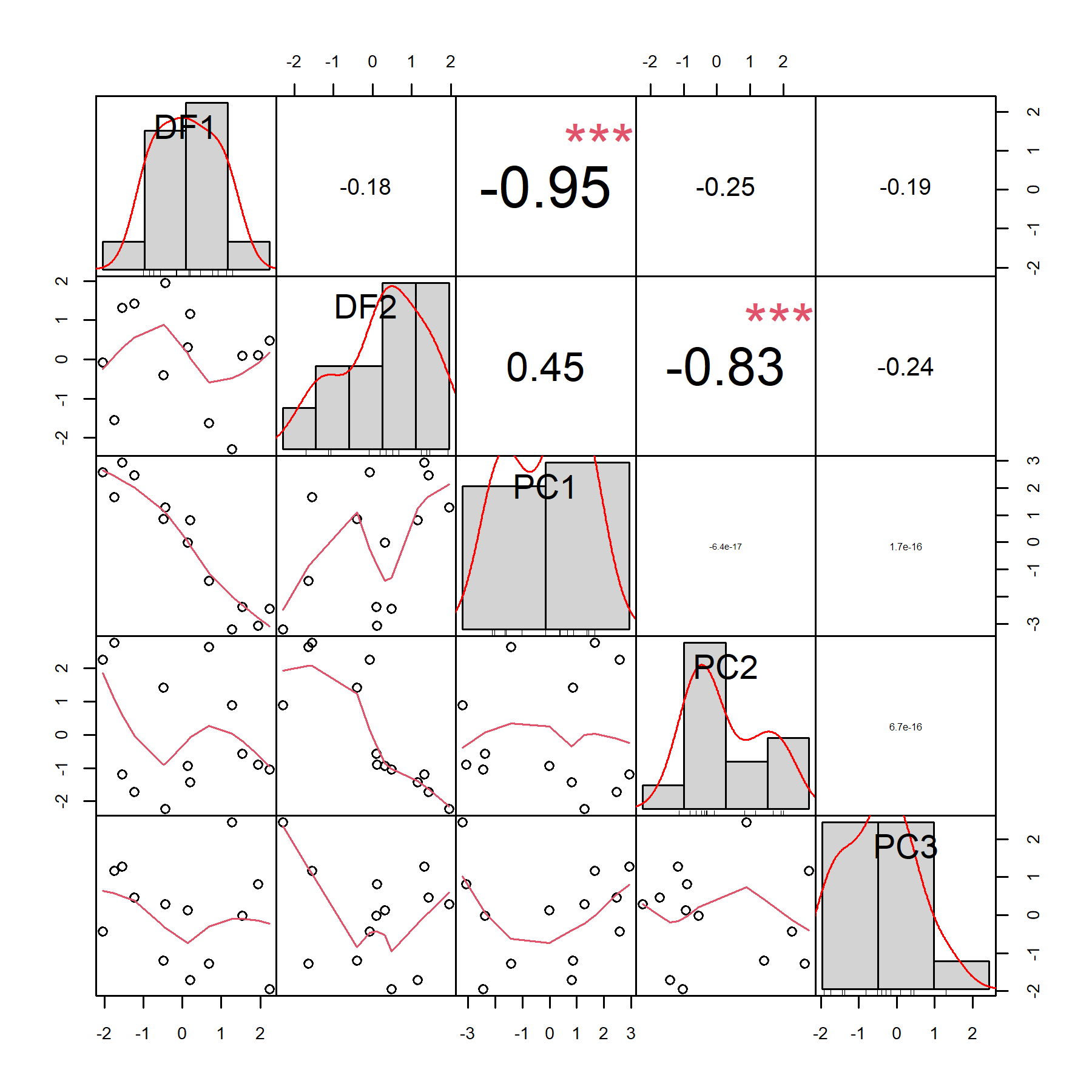

Supplement: S4 Fig — (TIF) [file pone.0251638.s010.tif]
